# Mitophagy Promotes Hair Regeneration by Activating Glutathione Metabolism

**DOI:** 10.34133/research.0433

**Published:** 2024-08-01

**Authors:** Dehuan Wang, Jingwei Jiang, Mengyue Wang, Ke Li, Huan Liang, Nian’ou Wang, Weiwei Liu, Miaomiao Wang, Siyi Zhou, Man Zhang, Yang Xiao, Xinyu Shen, Zeming Li, Wang Wu, Xia Lin, Xiao Xiang, Qiaoli Xie, Wanqian Liu, Xun Zhou, Qu Tang, Wei Zhou, Li Yang, Cheng-Ming Chuong, Mingxing Lei

**Affiliations:** ^1^ Key Laboratory of Biorheological Science and Technology of Ministry of Education and 111 Project Laboratory of Biomechanics and Tissue Repair, College of Bioengineering, Chongqing University, Chongqing 400044, China.; ^2^ Shenzhen Accompany Technology Cooperation, Ltd, Shenzhen 518000, China.; ^3^ Three Gorges Hospital, Chongqing University, Chongqing 404000, China.; ^4^ Department of Dermatology and Cosmetology, The First Affiliated Hospital of Chongqing College of Traditional Chinese Medicine, Chongqing 400021, China.; ^5^ Chongqing Key Laboratory of Translational Research for Cancer Metastasis and Individualized Treatment, Chongqing University Cancer Hospital, Chongqing 400030, China.; ^6^ Department of Pathology, Keck School of Medicine, University of Southern California, Los Angeles, CA 90033, USA.

## Abstract

Mitophagy maintains tissue homeostasis by self-eliminating defective mitochondria through autophagy. How mitophagy regulates stem cell activity during hair regeneration remains unclear. Here, we found that mitophagy promotes the proliferation of hair germ (HG) cells by regulating glutathione (GSH) metabolism. First, single-cell RNA sequencing, mitochondrial probe, transmission electron microscopy, and immunofluorescence staining showed stronger mitochondrial activity and increased mitophagy-related gene especially Prohibitin 2 (Phb2) expression at early-anagen HG compared to the telogen HG. Mitochondrial inner membrane receptor protein PHB2 binds to LC3 to initiate mitophagy. Second, molecular docking and functional studies revealed that PHB2-LC3 activates mitophagy to eliminate the damaged mitochondria in HG. RNA-seq, single-cell metabolism, immunofluorescence staining, and functional validation discovered that LC3 promotes GSH metabolism to supply energy for promoting HG proliferation. Third, transcriptomics analysis and immunofluorescence staining indicated that mitophagy was down-regulated in the aged compared to young-mouse HG. Activating mitophagy and GSH pathways through small-molecule administration can reactivate HG cell proliferation followed by hair regeneration in aged hair follicles. Our findings open up a new avenue for exploring autophagy that promotes hair regeneration and emphasizes the role of the self-elimination effect of mitophagy in controlling the proliferation of HG cells by regulating GSH metabolism.

## Introduction

**Fig. 1.**
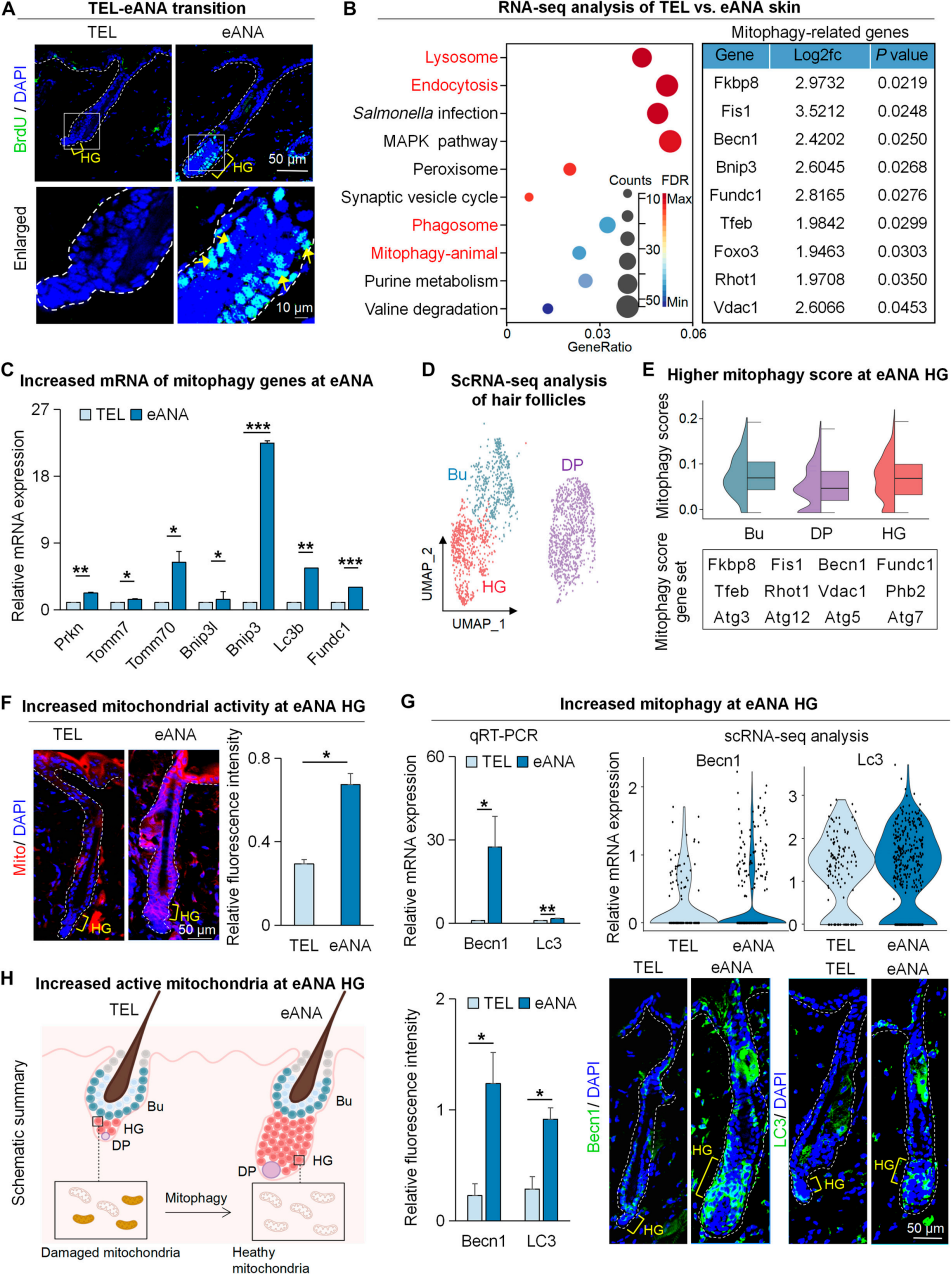
Increased mitophagy activity at early-anagen HG. (A) BrdU immunostaining for hair follicles during the transition from telogen to early anagen. Scale bars, 50 μm and 10 μm. (B) RNA-seq compares gene expression between telogen and early-anagen HFSCs. KEGG analysis shows the lysosome, endocytosis, phagosome, and mitophagy signaling pathways enriched in differentially expressed genes (DEGs) of telogen and early-anagen HFSCs (left). The table shows genes expressed in mitophagy pathways (right). (C) Quantitative RT-PCR shows mitophagy pathway genes that are differentially expressed at telogen (TEL) versus early anagen (eANA). *N* = 3, ***P < 0.001, **P < 0.01, *P < 0.05. (D) UMAP plots of Bu, HG, and DP clusters by unbiased clustering. (E) Half VlnPlot displays mitophagy scores of Bu, HG, and DP clusters. (F) Mitochondrial probe shows mitochondrial activity at telogen and early anagen. Scale bars, 50 μm. *N* = 3, *P < 0.05. (G) Quantitative RT-PCR, VlnPlot, and immunofluorescence staining show the expression of Becn1 and Lc3 in HG. Scale bars, 50 μm. *N* = 3, *P < 0.05. (H) Schematic of hair regeneration. During the transition from telogen to early anagen of the hair follicle, mitophagy activity is elevated in HG, which may drive hair regeneration.

Hair follicles as small epithelial organs of the skin maintain hair regeneration during repeated hair cycles that are divided into telogen, anagen, and catagen [1]. The hair germ (HG) is a small cell cluster located between the bulge and dermal papilla (DP). During the transition of the hair cycle from telogen to anagen, the HG first senses signals from the microenvironment and proliferates earlier than the bulge cells, providing fuel and being the main contributor to the initial steps of hair regeneration (Fig. 1A and Fig. S1A) [2]. Conceivably, this process is energy consumption that involves extensive metabolism. However, the metabolic mechanisms involved in hair regeneration still require further exploration.

Metabolism is involved in hair follicle stem cell (HFSC) development and hair regeneration [3]. Lactate dehydrogenase activity accelerates HFSC activation and the hair cycle [4]. Kim et al. [5] showed that glutamine metabolism is inhibited at the end of anagen follicle growth, allowing progenitor cells to return to the hypoxic ecological niche, restoring the stem cell state and regenerating bulge for long-term maintenance of HFSCs. Glutathione (GSH) influences stem cell function and differentiation. Down-regulation of GSH metabolism enhances and stabilizes the stem cell differentiation process [6]. Stem cells with higher GSH content have greater stemness, metastasis, and proliferation [7]. Increased expression of glutamate-cysteine ligase catalytic subunit (GCLC) and solute carrier family 7 member 11 (SLC7A11) can regulate GSH metabolism, promote GSH synthesis, and markedly increase the proliferation of Waldenstrom macroglobulinemia cells [8]. This suggests that GSH plays an important role in the differentiation of stem cells and the proliferation of cells. Metabolism is the energy state of cells. The metabolic process involves the activity of upstream macromolecules such as DNA and proteins. How metabolism is initiated during hair regeneration remains elusive.

Autophagy is the main intracellular degradation system, through which cytoplasmic substances are transported to lysosomes and degraded [7]. Autophagy, as a dynamic recycling system, generates new building blocks and energy for cell renewal and homeostasis [8]. Applying small molecules that activate autophagy to the back of mice can promote hair regeneration [9], but the mechanism by which autophagy promotes hair regeneration requires further investigation. Mitophagy is a selective autophagy specifically used for the self-elimination of damaged mitochondria. The self-elimination effect of mitophagy promotes the regeneration of skeletal muscle cells [10], self-renewal, differentiation, and aging of stem cells [11], as well as maintaining cell viability [12,13]. Transmission electron microscopy (TEM) showed that the mitochondria of hair follicle cells in the anagen are thinner and longer than those in the telogen, while the mitochondria in the telogen are more swollen, and the mitochondria in the anagen have more abundant cristae [11]. This suggests that mitochondria have different activities during hair cycling. Whether and how mitophagy regulates hair regeneration remains elusive.

During mitochondrial depolarization or proteasome-dependent outer membrane rupture, Prohibitin 2 (PHB2) binds to LC3 through the LC3 interaction region domain, participating in targeted mitophagy degradation [14]. PHB2 plays an important role in maintaining mitochondrial metabolism and repairing mitochondrial damage. Overexpression of PHB2 can activate mitophagy, restore mitochondrial activity, enhance myocardial cell viability, and inhibit endotoxin-induced mitochondrial dysfunction in myocardial cells [15]. The reduction of PHB2 inhibits the binding of PHB2 to LC3, inhibits mitophagy, exacerbates the loss of dopaminergic neurons, and accelerates the progression of Parkinson’s disease [16].

In astrocytes, mitophagy is induced by mitochondrial damage due to a series of effects, and downstream regulation leads to changes in GSH content [17]. Similarly, the knockdown (KD) of the mitophagy gene in HeLa cells resulted in decreased mitochondrial membrane potential and substantially reduced GSH content [17]. To explore the role of mitophagy in hair regeneration, we analyzed the mouse hair follicles in the telogen and early anagen and observed increased mitophagy activity at early-anagen HG. Functional research using hair depilation, hair shaving, and skin organoid models showed that HG cells first initiate mitophagy to eliminate mitochondria damaged potentially by apoptosis during catagen. Mitophagy promotes hair cycle transition and accelerates hair regeneration by regulating the GSH metabolism that supplies energy for HG cell proliferation. The aged HG cells can also be reactivated to regenerate upon the activation of mitophagy or GSH metabolism. Therefore, our study uncovered how mitophagy regulates hair regeneration by regulating GSH metabolism, paving a new way to solve the problem of hair loss that affects people’s lives and psychology.

## Results

### Elevated mitophagy activities in HG during the telogen-to-anagen transition

To investigate the molecular basis of hair regeneration during the transition from telogen to anagen, we performed RNA-seq analysis of telogen and early-anagen HFSCs that include bulge stem cells and HG cells. Kyoto Encyclopedia of Genes and Genomes (KEGG) enrichment analysis revealed that lysosome and mitochondrion pathways were highly enriched in early-anagen HFSCs (Fig. 1B and Fig. S1B). RNA-seq and quantitative reverse transcription polymerase chain reaction (qRT-PCR) showed that the expression of mitophagy genes was significantly increased in early anagen compared to telogen skin (Fig. 1B and C and Fig. S1B and C), indicating that mitophagy ability may be enhanced in hair follicles at early anagen. To further investigate the changes in mitophagy-related gene expression during hair regeneration, we selected the 3- and 4-week mice dorsal skin that represent the telogen and early anagen, respectively, and did single-cell RNA sequencing (scRNA-seq). We applied Seurat to reduce the dimensionality of scRNA-seq results and divided all cells into 28 clusters (Fig. S1D and E) using marker genes. We used scRNA-seq to extract the early-anagen Bu, HG, and DP (Fig. 1D), which are the effectors of hair regeneration to compare the mitophagy scores using a gene set, and found that the mitophagy scores were highest in HG of the early-anagen hair follicle (Fig. 1E).

We next used a mitochondrial probe that specifically labels biologically active mitochondria in the cell detects the mitochondrial membrane potential and observed the highest mitochondrial activity at early-anagen HG compared to other regions (Fig. 1F). ScRNA-seq, qRT-PCR, immunofluorescence staining, and statistical analysis revealed that the mitophagy-related genes Becn1 and Lc3 were indeed highly expressed at early-anagen HG compared to the telogen HG (Fig. 1G). These results imply that during the transition from telogen to early anagen, mitophagy activity is elevated in HG, which may drive hair regeneration (Fig. 1H).

### Phb2 responds to mitophagy activation at early-anagen HG

**Fig. 2.**
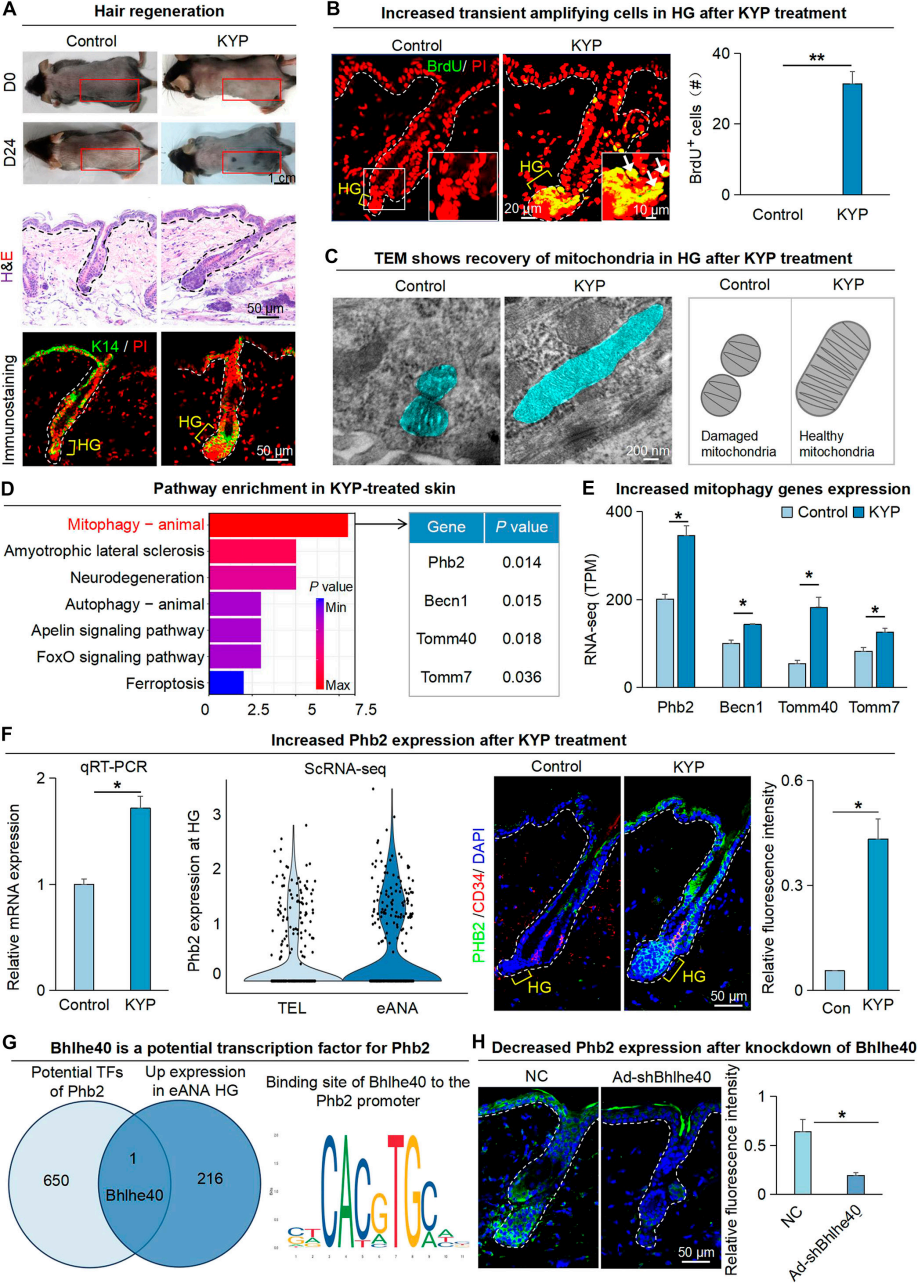
Increased expression of Phb2 at early-anagen HG. (A) Dissection microscopy, H&E staining and K14 immunofluorescence staining of hair follicles show that KYP induces hair regeneration. D0 represents the 0th day after shaving. Scale bars, 1 cm and 50 μm. (B) BrdU immunofluorescence staining shows that KYP promotes the proliferation of HG cells. The white arrow represents the Brdu^+^ cell. Scale bars, 20 μm and 10 μm. *N* = 3, **P < 0.01. (C) TEM shows that KYP promotes the recovery of mitochondrial activity in hair follicles. Scale bars, 200 nm. (D) RNA-seq compares gene expression in hair follicles between control and KYP-treated groups. KEGG analysis shows the mitophagy signaling pathway enriched in DEGs of control and KYP-treated groups (left). The table displays the genes expressed in the mitophagy pathway (right). (E) Quantitative RT-PCR shows the mRNA expression of mitophagy pathway that are differentially expressed in control and KYP-treated groups. *N* = 3, *P < 0.05. (F) Quantitative RT-PCR, VlnPlot, and immunofluorescence staining show the expression of Phb2 in HG. Scale bars, 50 μm. *N* = 3, *P < 0.05. (G) Venn diagram shows the number of common TFs predicted by Phb2 and the genes up-regulated at early anagen HG (left). Potential binding sites for Bhlhe40 to the Phb2 promoter sequences (right). (H) Left: Representative immunofluorescence images show the expression of Phb2 from shBhlhe40 and NC groups. Right: Quantitative analysis of relative fluorescence intensity. *n* = 3, *P < 0.05.

We next shaved hairs on the dorsal skin of 7-week-old C57BJ/6 mice and treated the shaved skin with small molecules KYP-2047 or SMER28 that activate Becn1 (Fig. S2A). On the 24th day after application, hair regeneration was evident in the applied area of the dorsal skin, whereas no hair regeneration was observed in the control group, and this is verified by hematoxylin and eosin (H&E) staining and K14 immunofluorescence staining (Fig. 2A and Fig. S2B). Immunofluorescence staining showed that the transient amplifying cells (5-bromodeoxyuridine [BrdU^+^]) were significantly increased in HG after KYP treatment indicative of stem cell activation, while no proliferating cells were observed in the control group (Fig. 2B). TEM showed that the mitochondria of the hair follicles in the control group exhibit a discrete spherical structure. In contrast, the mitochondria of the hair follicles treated with KYP had an elongated morphology with complete and more abundant cristae (Fig. 2C). These results suggest that activation of Becn1 can activate mitophagy to remove unhealthy or damaged mitochondria, accelerating the telogen-to-anagen transition process during hair regeneration.

To investigate the molecular bases during this process, we performed bulk RNA-seq of the KYP-treated or control mice dorsal skin. KEGG enrichment analysis of genes up-regulated in the KYP-treated mice skin revealed that the mitophagy pathway genes were most significantly enriched (Fig. 2D and E). Comparing the changes of mitophagy genes therein, we observed that Prohibitin 2 (Phb2) ranks first among the up-regulated genes in the KYP-treated skin (Fig. 2E). ScRNA-seq, qRT-PCR, and immunofluorescence staining showed that the expression of Phb2 was significantly increased at early anagen and anagen HG compared to telogen HG (Fig. 2F and Fig. S2C to E). This may suggest that Phb2 responds to mitophagy to regulate hair regeneration.

To elucidate the molecular mechanisms underlying Phb2 induction in early-anagen HG, we employed the Signaling Pathways Project website to identify potential regulatory transcription factors (TFs) for Phb2, resulting in the identification of 651 potential TFs. Intersection analysis of these 651 TFs with 217 up-regulated genes in early-anagen HG obtained from scRNA-seq data was performed, and we observed enrichment of only 1 TF named Bhlhe40 in potential TFs of Phb2 (Fig. 2G). The JASPAR website predicted the binding site between Bhlhe40 and Phb2 with over 80% confidence. Previous research has highlighted the pivotal role of Bhlhe40 in cell differentiation and its regulation of mitophagy in stem cells [18,19]. Bulk RNA-seq and scRNA-seq revealed a significant up-regulation of Bhlhe40 expression in early-anagen HG compared to telogen HG (Fig. S2F). To verify the function of Bhlhe40 in hair regeneration, we further knocked it down using short-hairpin RNA (shRNA) in skin organoids. As expected, the KD of Bhlhe40 in epidermal cells significantly impaired their ability to induce hair regeneration from the skin organoids posttransplantation (Fig. S2G and H). Next, we packaged the shRNA into adenovirus and delivered it into the back of plucked mice by subcutaneous injection. qRT-PCR and immunostaining showed that KD of Bhlhe40 inhibited the expression of PHB2 compared with the nontargeting control (NC)-treated group (Fig. 2H and Fig. S2I). Based on these observations, we speculate that Bhlhe40 induces Phb2 expression in early-anagen HG, thereby facilitating the interaction between PHB2 and LC3 to instigate mitophagy, eliminating damaged mitochondria accumulated in telogen.

### PHB2 inhibition will block hair regeneration

**Fig. 3.**
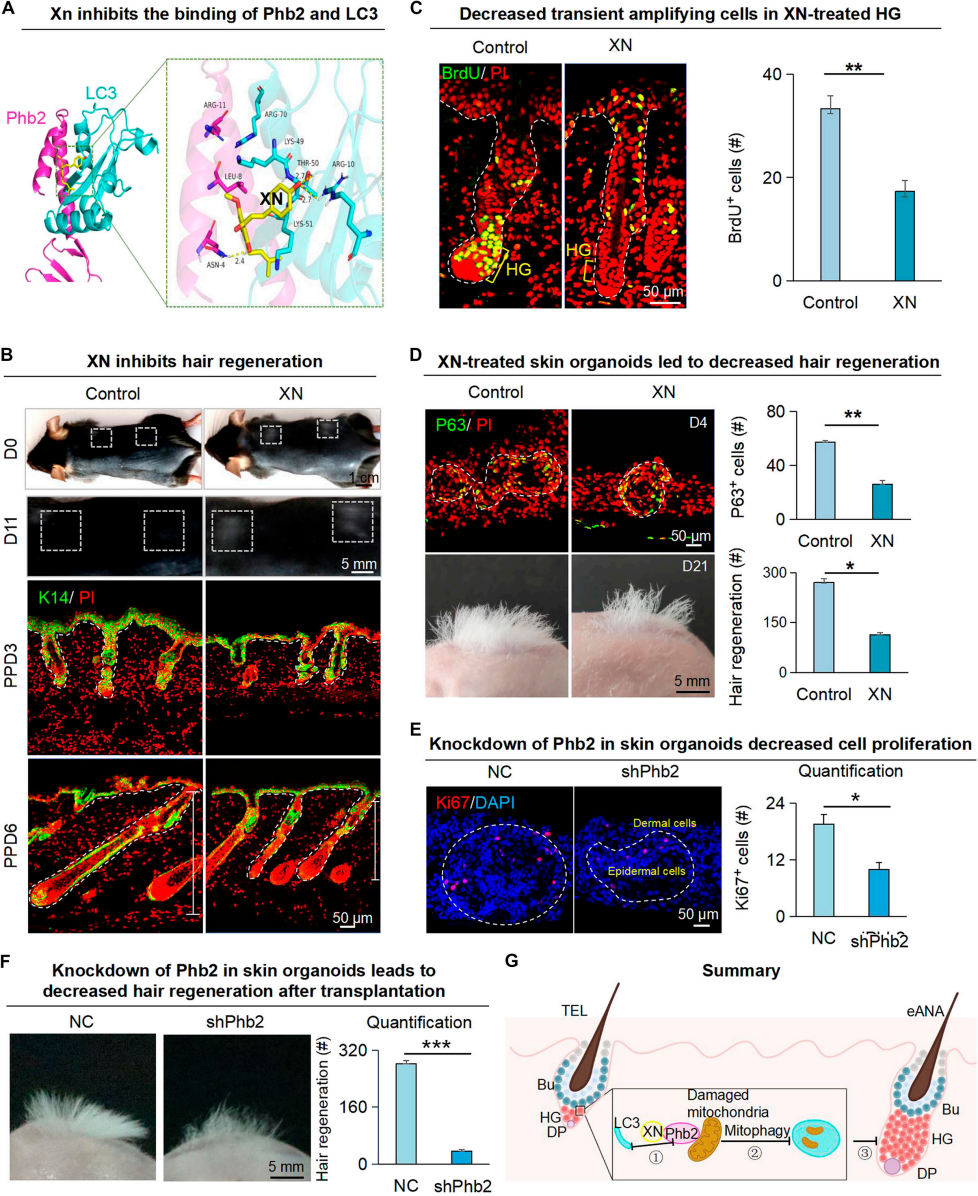
Inhibition of PHB2 and LC3 binding by XN inhibits hair regeneration. (A) PyMOL displays the molecular docking results of XN with LC3 and Phb2. (B) Dissection microscopy and K14 immunofluorescence staining of hair follicles show that XN inhibits hair regeneration at PPD3, PPD6, and PPD11. Scale bars, 1 cm, 5 mm, and 50 μm. (C) BrdU immunofluorescence staining shows that XN inhibits the proliferation of HG cells at PPD3. Scale bars, 50 μm. *N* = 3, **P < 0.01. (D) XN in skin organoids inhibits hair regeneration after transplantation. P63 immunofluorescence staining of skin organoid cultures of newborn mice cells shows the epidermal stem cells. Dissection microscopy and statistical analysis show hair regeneration after transplantation. Scale bars, 50 μm and 5 mm. *N* = 3, **P < 0.01, *P < 0.05. (E) Left: Representative immunofluorescence images of Ki67 in skin organoids from shPhb2 and NC groups. Dashed lines indicate epidermal cell clusters inside and dermal cells outside. Right: Quantitative analysis of Ki67^+^ cell numbers. *n* = 3, *P < 0.05. (F) Left: Representative images illustrating hair regeneration after skin organoid transplantation from the NC and shPhb2 groups. Right: Quantitative analysis of the number of hair follicles regenerated. *n* = 3, ***P < 0.001. (G) Schematic of XN inhibiting the binding of Phb2 and LC3 and inhibiting hair regeneration.

PHB2 is an important mitophagy receptor that is involved in targeting mitochondria for autophagic degradation. When mitochondrial depolarization and proteasome-dependent rupture of the outer membrane occur, PHB2 binds the autophagosome membrane-associated protein LC3 via the LC3 interaction region structural domain (Fig. S3A) [14], facilitating the generation of mitophagy and the removal of damaged mitochondria. xanthohumol (XN), a ligand of PHB2, inhibits autophagic degradation of mitochondria through association with PHB2 binding and inhibiting the binding of PHB2 to LC3, which in turn inhibits mitophagy (Fig. 3A).

Hair plucking on the back of mice induces hair follicles to transition from telogen to anagen [20,21]. We next used this model to test if PHB2-mediated mitophagy influences hair regeneration (Fig. S3B). Dissection microscopy, H&E staining, and immunofluorescence staining showed that most hair follicles entered anagen VI on the postplucking day 6 (PPD6) in the control group, while the hair follicles in the XN-treated group were still at anagen II or III on PPD6 with decreased Wnt activity (Fig. 3B and Fig. S3C and D), indicating that hair regeneration is delayed. Immunofluorescence staining showed no obvious BrdU^+^ cells in HG of the XN-treated group (Fig. 3C), suggesting that Phb2-mediated mitophagy influences HG proliferation.

We next used our established organoid model to test mitophagy and hair regeneration (Fig. S3E) [22]. We constructed the skin organoids by extracting the skin cells from newborn mice and added XN to the skin organoids. The results showed that compared to the control group, the XN-treated group resulted in a significant reduction of epidermal stem cells (P63^+^) in the organoid culture and a decrease in hair growth on the back of nude mice after transplantation (Fig. 3D). To confirm the functional role of PHB2, we performed shRNA-mediated KD of Phb2 in skin organoids and investigated its impact on hair regeneration (Fig. S3F). KD of Phb2 in epidermal cells using shRNA significantly reduced cell proliferation and impaired the ability to induce hair regeneration from skin organoids posttransplantation (Fig. 3E and F). This suggests that the blocking of PHB2-LC3 binding by XN or the KD of Phb2 hinders the self-elimination effect of mitophagy, which then inhibits hair regeneration (Fig. 3G).

### LC3 mediates mitophagy for hair regeneration

**Fig. 4.**
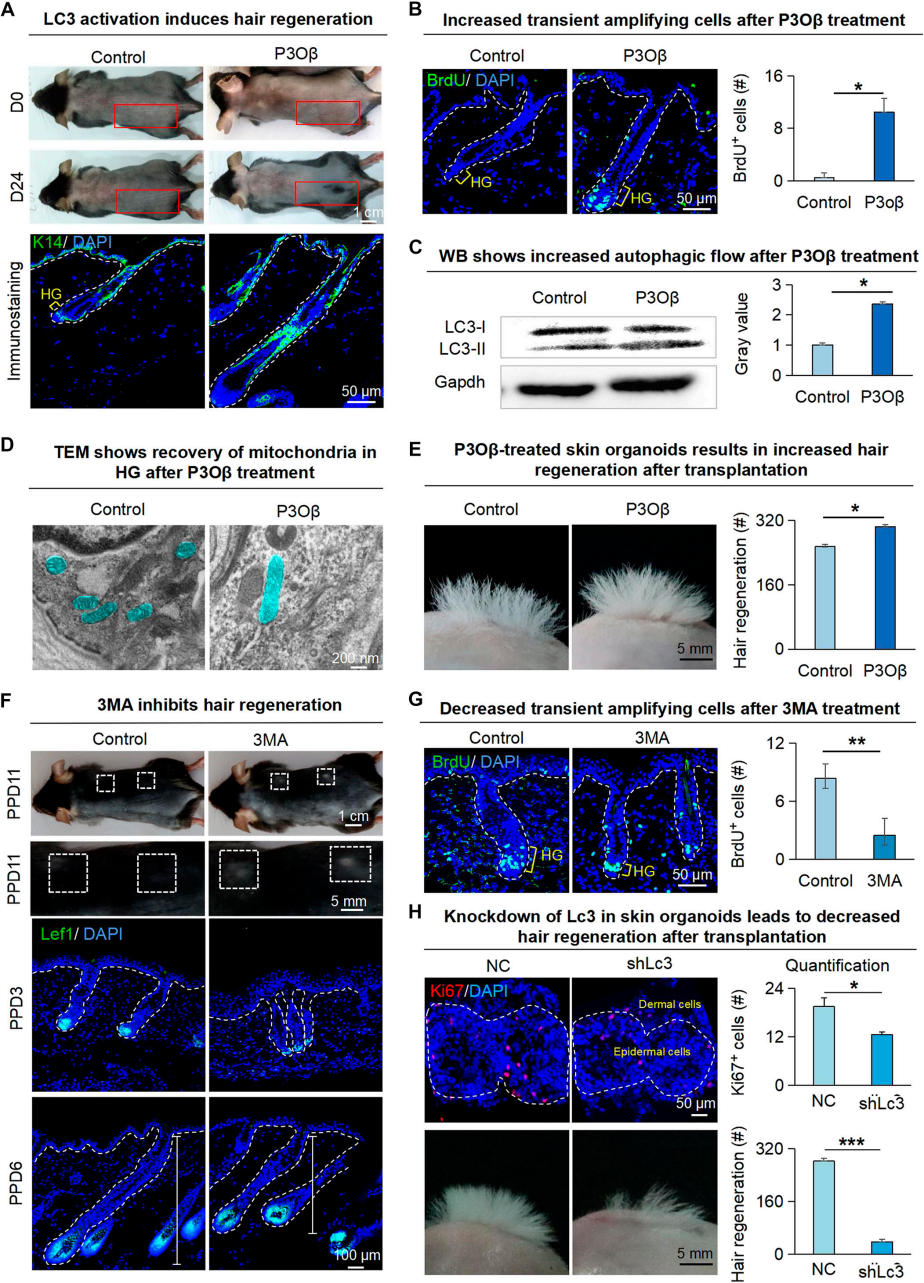
Lc3 mediated mitophagy to promote the proliferation of HG cells and hair regeneration. (A) Dissection microscopy, H&E staining, and K14 immunofluorescence staining of hair follicles show that P3Oβ induces hair regeneration. Scale bars, 1 cm and 50 μm. (B) BrdU immunofluorescence staining shows that P3Oβ promotes the proliferation of HG cells. Scale bars, 50 μm. *N* = 3, *P < 0.05. (C) Western blot and statistical analysis show that P3Oβ promotes the formation of autophagic flow from LC3-I to LC3-II. *N* = 3, *P < 0.05. (D) TEM shows that P3Oβ promotes the recovery of mitochondrial activity in hair follicles. Scale bars, 200 nm. (E) Dissection microscopy and statistical analysis show hair regeneration after transplantation. Scale bars, 5 mm. *N* = 3, *P < 0.05. (F) Dissection microscopy and Lef1 immunofluorescence staining of hair follicles show that 3MA inhibits hair regeneration at PPD3, PPD6, and PPD11. Scale bars, 1 cm, 5 mm, and 50 μm. (G) BrdU immunofluorescence staining shows that 3MA inhibits the proliferation of HG cells at PPD3. Scale bars, 50 μm. *N* = 3, **P < 0.01. (H) Left: Representative image showing Ki67 expression in skin organoids upon KD of Lc3 (upper). Representative images illustrating hair regeneration after skin organoid transplantation from the NC and shLc3 groups (lower). Right: Quantitative analysis of Ki67^+^ cell numbers (upper); quantitative analysis of the number of hair follicles regenerated (lower). *n* = 3, *P < 0.05, ***P < 0.001.

To explore how LC3 affects hair regeneration, we treated the backs of 7-week-old shaved mice with a small molecule that can activate LC3. Regenerated hairs can be observed in the pennogenin 3-O-beta-chacotrioside (P3Oβ)-treated group on the D24 (Fig. 4A). Dissection microscopy, H&E staining (Fig. S4A) and immunofluorescence staining (Fig. 4A and Fig. S4A) showed that the hair follicles in the P3Oβ-treated group entered anagen, while the control group was still in the telogen. BrdU staining indicated significantly more proliferating cells in HG of the P3Oβ-treated group compared with the control group (Fig. 4B). During autophagy, the cytoplasmic form of LC3-I is modified to LC3-II. Therefore, the amount of LC3-II is increased with the formation of autophagosomes [23]. Indeed, the western blot result showed a significant increase of LC3-II in the P3Oβ-treated group compared to the control group (Fig. 4C). TEM revealed that the mitochondrial morphology of the hair follicles in the P3Oβ-treated group was normal with intact cristae, while the mitochondria in the control group were in a folded shape (Fig. 4D). Transplantation of P3Oβ-treated skin organoids onto the back of the nude mice resulted in significantly more hair regeneration compared to the control (Fig. 4E).

We also verified whether LC3 is required for the hair regeneration process using a small molecule 3MA that inhibits LC3, to treat mice after dorsal hair plucking. Dissection microscopy (Fig. 4F), H&E staining (Fig. S4B), and immunofluorescence staining (Fig. 4F and Fig. S4B) showed that the hair regeneration was delayed in the 3MA-treated group on PPD6, with fewer BrdU^+^ cells in HG region on PPD3 compared to the control (Fig. 4G). Immunofluorescence staining showed that the 3MA-treated skin organoids have fewer epidermal stem cells than in the control group (Fig. S4C). Transplantation of 3MA-treated skin organoids onto the back of the nude mice led to significantly fewer hair regeneration compared to the control (Fig. S4C). We also investigated the impact of LC3 on hair regeneration in skin organoids. KD of Lc3 in skin organoids using shRNA significantly reduced cell proliferation and impaired hair regeneration postorganoid transplantation (Fig. 4H). Our results verified that LC3 is required for hair cycle transition.

### LC3 promotes hair regeneration by activating GSH metabolism

**Fig. 5.**
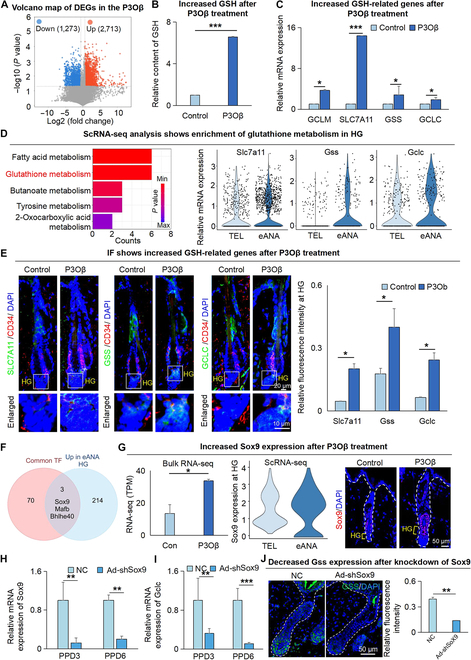
Lc3 regulates the GSH metabolism pathway in HG cells. (A) Volcano map compares gene expression in hair follicles between control and P3Oβ-treated groups. (B) The GSH assay kit determined the GSH content of control and P3Oβ-treated groups. (C) Quantitative RT-PCR shows the mRNA expression of the GSH metabolism pathway that is differentially expressed in the control and P3Oβ-treated groups. (D) ScRNA-seq compares gene expression in hair follicles between telogen and early anagen. KEGG analysis shows the GSH metabolism enriched in HG (left). VlnPlot shows the mRNA expression of GSH metabolism pathways that are differentially expressed in HG between telogen and early anagen. (E) Immunofluorescence staining of Slc7a11, Gss, and Gclc show the expression of HG in the control group and P3Oβ-treated group. Scale bars, 50 μm and 10 μm. *N* = 3, *P < 0.05. (F) Venn diagram shows the number of common TFs predicted by 4 genes related to GSH synthesis and the gene up-regulated at early-anagen HG. (G) Quantitative RT-PCR, VlnPlot, and immunostaining show the expression of Sox9 in HG. Scale bars, 50 μm. *N* = 3, *P < 0.05. (H) Quantitative RT-PCR shows the expression level of Sox9 from NC and Ad-shSox9 group at PPD3 and PPD6. *n* = 3, **P < 0.01. (I) Quantitative RT-PCR shows the expression of Gclc from NC and Ad-shSox9 group at PPD3 and PPD6. *n* = 3, **P < 0.01, ***P < 0.001. (J) Representative immunofluorescence images show the expression of Gss from Ad-shSox9 and NC groups. Right: Quantitative analysis of relative fluorescence intensity. Scale bars, 50 μm. *n* = 3, **P < 0.01.

It has been shown that autophagy affects cell metabolism [24,25]. To investigate if mitophagy influences metabolic processes during hair regeneration, we performed RNA-seq for the P3Oβ-treated and control group (Fig. 5A). KEGG enrichment analysis of enriched metabolic pathways indicated that significantly increased genes in the P3Oβ-treated skin are enriched in the GSH metabolism pathway (Fig. S5A). It is reasonable to speculate that hair regeneration with abundant cell proliferation requires pyrimidine and nucleotide metabolism, which showed significant enrichment in KEGG analysis (Fig. S5A), to support the DNA synthesis. Thus, we selected the GSH metabolism pathway to do further study. By using the GSH detection kit, we found the GSH content was significantly increased in the P3Oβ-treated group, with 6 to 8 times higher than the control group (Fig. 5B). RNA-seq analysis (Fig. S5B) and qRT-PCR (Fig. 5C) showed that the key genes Slc7a11, Gclm, Gss, and Gclc involved in the GSH metabolism pathway were significantly higher in the P3Oβ-treated group compared to the control group. The GSH and oxidized glutathione (GSSG) detection assay demonstrated a significant elevation in the GSH/GSSG ratio within P3Oβ, suggesting mitigation of oxidative stress (Fig. S5C). Glutathione reductase (GR) is a crucial enzyme within the cellular redox system responsible for maintaining cellular levels of reduced GSH [26]. GR Activity Assay Kit detection assay showed that compared with the control group, the GR enzyme activity in P3Oβ was significantly increased, indicating that the oxidative stress behavior of cells was inhibited.

To analyze the key GSH metabolism-related genes involved in hair regeneration, we used RNA-seq analysis (Fig. S5D) and a GSH detection kit (Fig. S5E), which revealed that GSH metabolism was significantly enriched in the early-anagen skin. ScRNA-seq analysis by isolating HG cells revealed that the GSH metabolism pathway genes were significantly enriched (Fig. 5D), and the expression of GSH-metabolism-pathway-related genes was higher at early anagen compared to telogen HG (Fig. 5D and Fig. S5F). Immunofluorescence staining verified that SLC7A11, GSS, and GCLC were highly expressed at early-anagen HG in the P3Oβ-treated group compared with the control group (Fig. 5E). On the contrary, qRT-PCR revealed that the expression of Slc7a11 and Gclm was significantly decreased in the 3MA-treated group compared with the control group (Fig. S5F). Immunofluorescence staining showed that SLC7A11 was significantly decreased in the 3MA-treated hair follicles on PPD3, compared to the control group (Fig. S5G). The above results suggest that LC3 regulates hair regeneration by modulating the GSH metabolism in the HG region.

To explore the potential molecular mechanisms underlying the regulation of GSH metabolism by mitophagy, Signaling Pathways Project predicted TFs associated with 4 up-regulated genes involved in GSH synthesis (Gclm, Gss, Gclc, and Slc7a11) in P3Oβ compared to controls. This analysis revealed a shared pool of 73 candidate TFs for these genes (Fig. S5H). Intersection of these TFs with 217 up-regulated genes in early-anagen HG led to the identification of 3 TFs (Sox9, Bhlhe40, and Mafb) that potentially regulate GSH synthesis and are elevated in early-anagen HG (Fig. 5F). Bulk RNA-seq and scRNA-seq showed a more significant up-regulation of Sox9 in early-anagen HG and a significantly higher expression of Sox9 in the P3Oβ group as compared to control (Fig. 5G and Fig. S5I). The JASPAR website predicted the binding site between Sox9 and Gss with over 80% confidence (Fig. S5J). Sox9, a recognized marker gene for HFSCs critical for their formation and maintenance, has been implicated in promoting hair regeneration [27,28]. Indeed, KD of Sox9 in epidermal cells using shRNA significantly impaired the ability to induce hair regeneration after skin organoid transplantation (Fig. S5K and L). To further assess the function of Sox9, we delivered shSox9 by adenovirus into the back of plucked mice by subcutaneous injection. Immunostaining and qRT-PCR showed that KD of Sox9 significantly decreased the expression of Gclc and Gss/GSS compared to the NC-treated group (Fig. 5H to J).

We hypothesize that mitophagy preserves mitochondrial function during hair regeneration by eliminating damaged mitochondria, thereby influencing the expression and function of Sox9, promoting the synthesis of GSH and cell proliferation within HG, and ultimately facilitating hair regeneration.

### GSH promotes hair regeneration

**Fig. 6.**
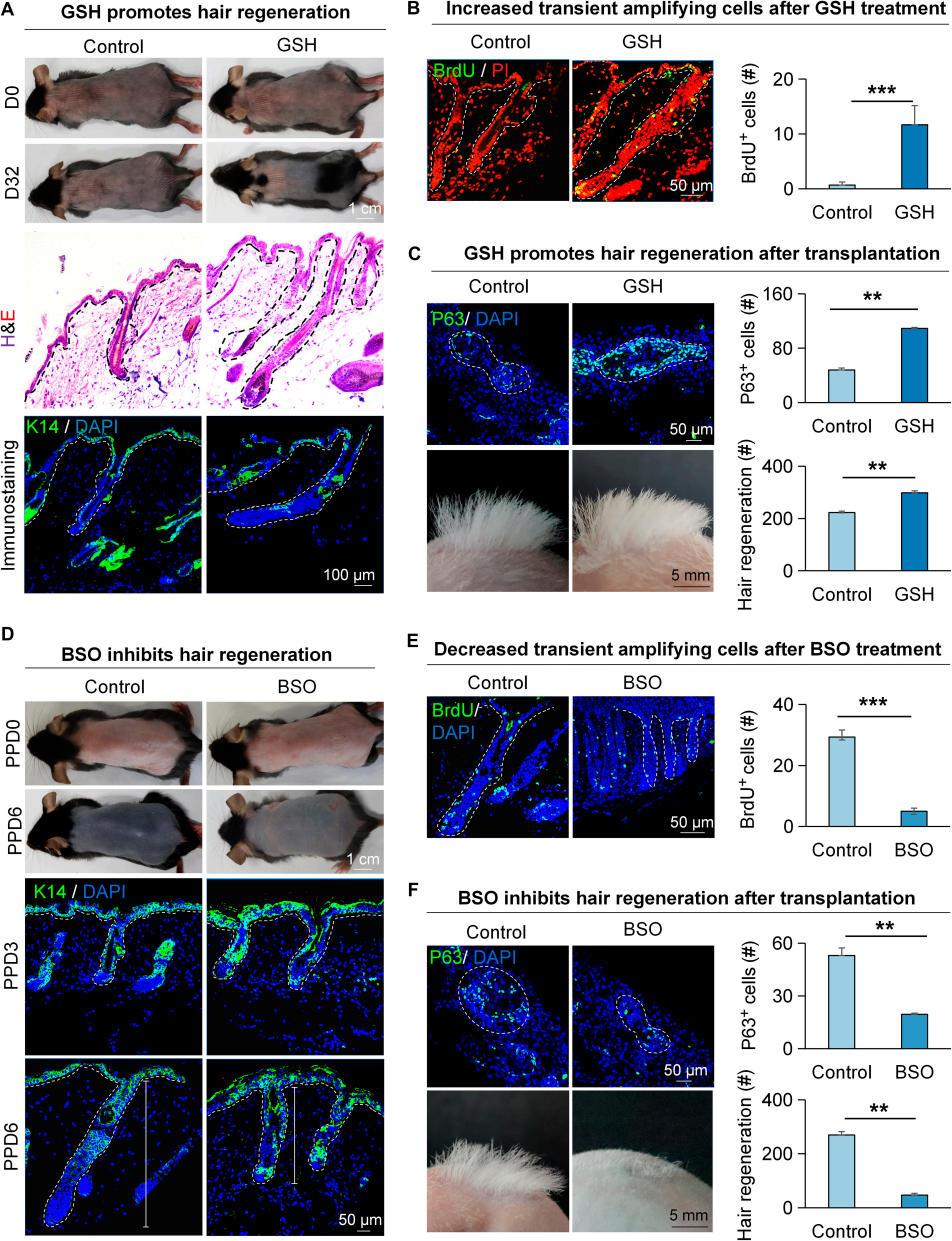
GSH promotes the proliferation of HG cells and hair regeneration. (A) Dissection microscopy, H&E staining, and K14 immunofluorescence staining of hair follicles show that GSH induces hair regeneration. Scale bars, 1 cm and 50 μm. (B) BrdU immunofluorescence staining shows that GSH promotes the proliferation of HG cells. Scale bars, 50 μm. *N* = 3, ***P < 0.001. (C) GSH in skin organoids promotes hair regeneration after transplantation. P63 immunofluorescence staining of skin organoid cultures of newborn mice cells shows the epidermal stem cells. Dissection microscopy and statistical analysis show hair regeneration after transplantation. Scale bars, 50 μm and 5 mm. *N* = 3, **P < 0.01. (D) Dissection microscopy and K14 immunofluorescence staining of hair follicles show that BSO Inhibits hair regeneration at PPD3 and PPD6. PPD0 represents the 0th day after plucking. Scale bars, 1 cm, 5 mm, and 50 μm. (E) BrdU immunofluorescence staining shows that BSO inhibits the proliferation of HG cells at PPD3. Scale bars, 50 μm. *N* = 3, ***P < 0.001. (F) BSO in skin organoids inhibits hair regeneration after transplantation. P63 immunofluorescence staining of skin organoid cultures of newborn mice cells shows the epidermal stem cells. Dissection microscopy and statistical analysis show hair regeneration after transplantation. Scale bars, 50 μm and 5 mm. *N* = 3, **P < 0.01.

To verify if GSH affects hair regeneration, we intraperitoneally injected GSH into the 7-week-old shaved mice. The GSH content detection kit showed that the GSH content was significantly higher in the GSH-treated group compared to the control group (Fig. S6A). Dissection microscopy, H&E staining, and immunofluorescence staining showed that the hair regeneration was accelerated in the GSH-treated group, with significantly more BrdU^+^ cells in the hair follicle compared to the control (Fig. 6A and B and Fig. S6B). Immunofluorescence staining revealed significantly more P63^+^ cells in the GSH-treated D4 skin organoid compared to the control group (Fig. 6C). Transplantation of GSH-treated skin organoids onto the back of nude mice resulted in significantly more hair regeneration compared to the control (Fig. 6C).

Conversely, we intraperitoneally injected DL-buthionine-(S,R)-sulfoximine (BSO), which is a small molecule that effectively inhibits GSH synthesis [29], to treat mice after dorsal hair plucking and observed that BSO treatment led to decreased GSH content (Fig. S6C), delayed hair regeneration (Fig. 6D and Fig. S6D), and decreased BrdU^+^ proliferation cells in HG (Fig. 6E). Transplantation of BSO-treated skin organoids with fewer P63^+^ cells onto the back of nude mice led to significantly fewer hair regeneration compared to the control (Fig. 6F). These results indicate that GSH activates cell proliferation in hair follicles and promotes hair regeneration.

### P3Oβ and GSH promote hair regeneration in aged mice

**Fig. 7.**
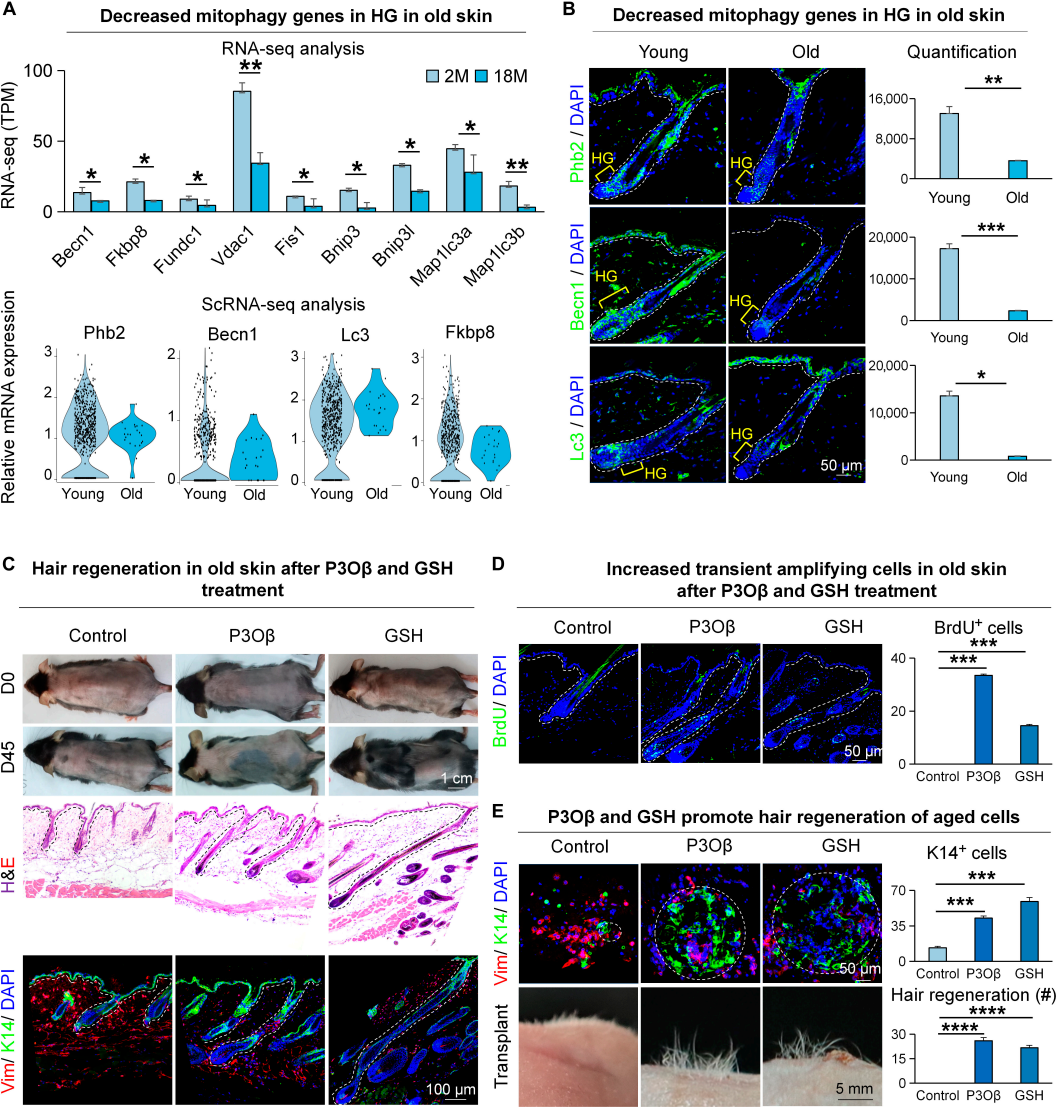
P3Oβ and GSH promote hair regeneration in aged mice. (A) RNA-seq shows the mRNA expression of the mitophagy pathway that is differentially expressed in young and aged mice. VlnPlot shows the mRNA expression of the mitophagy pathway that is differentially expressed in young and aged mice. *N* = 3, **P < 0.01, *P < 0.05. (B) Immunofluorescence of Phb2, Becn1, and Lc3 shows the expression of HG in young and aged mice. Scale bars, 50 μm. *N* = 3, ***P < 0.001, **P < 0.01, *P < 0.05. (C) Dissection microscopy, H&E staining, and K14 immunofluorescence staining of hair follicles show that P3Oβ and GSH induce hair regeneration in aged mice. Scale bars, 1 cm and 50 μm. (D) BrdU immunofluorescence staining shows that P3Oβ and GSH induce the proliferation of HG cells in aged mice. Scale bars, 50 μm. *N* = 3, ***P < 0.001. (E) P3Oβ and GSH in skin organoids induce hair regeneration after transplantation. K14 and Vimentin immunofluorescence staining of skin organoid cultures of adult mice cells shows the formation of aggregates. Dissection microscopy and statistical analysis show hair regeneration after transplantation. Scale bars, 50 μm and 5 mm. *N* = 3, ****P < 0.0001, ***P < 0.001.

Studies in mice and humans have shown that aging leads to decreased mitochondrial function and impaired mitophagy [30–32], and this may lead to decreased hair regeneration in aged mice. To verify this, we used RNA-seq and qRT-PCR, which revealed a significant decrease in mitophagy-related gene expression in aged mice compared to young mice (Fig. 7A and Fig. S7A). ScRNA-seq analysis showed that the expression of mitophagy-related genes was down-regulated in the hair follicles of the aged mice compared with the young mice (Fig. 7A and Fig. S7B). Immunofluorescence staining similarly revealed that the expression of PHB2, BECN1, and LC3 was significantly decreased in HG of the aged mice compared to HG of the young mice (Fig. 7B). To investigate whether activation of mitophagy would promote hair regeneration in aged mice, we applied P3Oβ onto the dorsal skin or intraperitoneally injected GSH into 18-month-old C57 mice after shaving. The results showed that hairs were regenerated on the backs of mice in the P3Oβ- and GSH-treated group on day 45 after drug administration (Fig. 7C). H&E staining and immunofluorescence staining showed that the hair regeneration entered anagen in the P3Oβ- and GSH-treated group, with significantly more BrdU^+^ cells in the hair follicles, compared to the control (Fig. 7C and D). Immunofluorescence staining for K14, Vimentin, and Collagen XVII revealed significantly more epidermal stem cells in the P3Oβ- and GSH-treated D4 skin organoid compared to the control group that only forms tiny epidermal aggregates (Fig. 7E and Fig. S7D). Transplantation of P3Oβ- or GSH-treated skin organoids onto the back of nude mice led to significantly more hair regeneration compared to the control (Fig. 7E). These results imply that activation of the mitophagy or GSH metabolism pathway in aged cells promotes hair regeneration.

## Discussion

In this study, we found that the selective self-elimination effect of mitophagy is sufficient and necessary for telogen-to-anagen transition in hair follicles. Compared with the hair follicles at telogen, mitochondria were more intact and healthy, with stronger mitochondrial activity at early-anagen HG. Mitophagy-related genes such as Phb2, Lc3, and Becn1 were up-regulated during the telogen-to-anagen transition. Mitophagy is an energy-consuming process that requires the initiation of energy metabolism to maintain cellular function. We found that during the hair follicle cycle transition, mitophagy further regulates GSH metabolism, contributing to HG proliferation followed by hair regeneration. We also showed that activation of mitophagy results in hair regeneration in aged mice and skin organoid cultures. Since hair follicle apoptosis occurs during catagen and the activity of the hair follicle is minimized during telogen [33], our study proposes a new principle that mitophagy maintains intracellular mitochondrial homeostasis and repairs the cell through selective self-elimination of the damaged mitochondria accumulated during catagen and telogen, thus is required for hair regeneration.

### Mitophagy-mediated self-elimination is essential for tissue regeneration

The present study demonstrated that mitophagy is sufficient and required for hair regeneration. First, we found that when mitophagy was activated by using the Becn1 agonist KYP-2047 and the LC3 agonist P3Oβ, hair regeneration was accelerated (Figs. 2A and 4A). Second and on the contrary, inhibiting the binding of PHB2 and LC3 with XN or inhibiting the function of LC3 with 3MA to inhibit mitophagy leads to delayed hair regeneration (Figs. 3B and 4F). Third and importantly, we showed that activation or inhibition of mitophagy in skin organoid culture also influences epidermal stem cell behavior which influences hair regeneration upon transplantation. This infers that mitophagy entails hair regeneration.

Indeed, the ability of selective self-elimination is essential for cell proliferation and tissue regeneration. Mitophagy promotes the proliferation of liver cells by repairing mitochondrial function [34]. During carcinogenesis, cancer cells can utilize the mitophagy pathway to increase their metabolic demand and resistance to cell death, leading to increased proliferation and invasive capacity of cancer cells [35]. In the process of skin wound healing, the increase of mitophagy induces the proliferation and migration of dermal cells and accelerates the wound healing process [36]. Therefore, the self-elimination effect of mitophagy influences tissue regeneration.

### GSH metabolism promotes the proliferation of HG cells

Stem cells change metabolism during homeostasis and proliferation [37], and glutamine metabolism is constantly shifted among HFSCs during their exit from their bulge niche to produce outer root sheaths during anagen progression, as well as the return of a subset of outer root sheath progeny to the bulge niche to restore the stem cell state to adapt to and regain reversibility of the HFSC fate during catagen [5]. Induction of cell stemness and maintenance inhibits mitochondrial oxidative phosphorylation while increasing glycolysis, which can accelerate stemness induction and accelerate hair regeneration by stimulating the progression of the hair cycle toward anagen [38]. GSH is a tripeptide synthesized from glutamate, cysteine, and glycine, which plays an important role in cell signaling and antioxidant processes [39]. GSH depletion in rats affects mitochondrial function during liver regeneration, resulting in diminished liver regeneration [40]. We observed that increased mRNA and protein expression levels of Slc7a11, Gclc, and Gss can promote the synthesis of GSH and accelerated telogen-to-anagen transition when mice were supplemented with GSH (Fig. 6A to E), whereas inhibition of GSH by intraperitoneal injection of BSO inhibited hair regeneration (Fig. 6F to J). Particularly, we observed significantly more proliferation cells in HG of GSH-treated mice, suggesting that GSH supplies the proliferation of HG cells and promotes hair regeneration.

### Mitophagy and GSH enhance hair regeneration in aged hair follicles

Tissues and organs undergo structural and functional declines in the aging process, with decreased regenerative ability [41]. Timely self-elimination of damaged mitochondria from cells through mitophagy is an important quality control mechanism for cell homeostasis and survival during injury and repair [42]. It is widely recognized that with aging, cell function, and metabolism are affected and the self-repair capacity of cells decreases [43]. In addition to intrinsic or extrinsic silenced stem cell activation signals [1], another possibility for decreased hair regeneration might be the degressive ability of self-elimination of damaged mitochondria. In the present study, we found that the level of mitophagy was decreased in aged hair follicles (Fig. 7A and B). Activation of mitophagy accelerates the transition of hair follicles from telogen to anagen (Fig. 7C and D). This suggests that activation of mitophagy rescues the mitochondrial dysfunction in aged hair follicles, restores the self-repairing ability of aged hair follicles, activates the proliferation of HFSCs, and promotes hair regeneration.

In conclusion, our study used scRNA-seq analysis, animal models, skin organoid culture and transplantation to show that PHB2 initiates mitophagy by binding to LC3 and selectively self-eliminates damaged mitochondria, promoting hair regeneration in mice. During catagen, hair follicle cells undergo continuous apoptosis and degradation upwards, while HFSCs remain quiescent in telogen. Consequently, damaged mitochondria may accumulate in telogen hair follicles. Upon entering early anagen, hair follicles initiate mitophagy to eliminate damaged mitochondria, restore cellular homeostasis, and initiate hair regeneration. Furthermore, mitophagy regulates the expression of SLC7A11, GSS, and GCLC, promoting GSH synthesis that enhances the proliferation of HG cells and facilitates hair regeneration in mice. We observed decreased expression of mitophagy-related genes in aging hair compared to young hair, potentially due to the prolonged hair cycle in older mice. Activation of LC3 or supplementation with GSH was found to shorten the hair cycle and promote hair regeneration in aged mice, suggesting potential clinical applications. This study innovates the understanding of the significance of the self-elimination effect in metabolic regulation and tissue regeneration, and it also identified several small-molecule drugs such as KYP-2047, P3Oβ, and GSH that can promote hair regeneration. This work provides a new idea for the development of hair regeneration drugs and opens up a new way to solve the problem of hair loss.

## Materials and Methods

### Mice

The animal experiment protocol was approved by the Chongqing University Animal Experimentation Ethics Committee. C57BL/6 mice (7/8-week-old females) were purchased from Chongqing Enbi Biotechnology Co. Ltd., and nude mice (8-week-old males) were purchased from Beijing Viton Lihua Laboratory Animal Technology Company. Three to 5 mice were housed in each cage under the following control conditions: a stable temperature of 25±1°C, a 12-h light/12-h dark cycle, and the availability of food and water. Experimental mice of the same sex were randomly assigned to experimental groups.

### Small-molecule administration

After shaving the backs of 7-week-old C57 mice, 100 μl of 400 μM KYP-2047 (T8657, Taojiao, China), 8 mM SMER28 (T3155, Taojiao, China), 200 μM Pennogenin 3-O-beta-chacotrioside (T8166, Taojiao, China), or dimethyl sulfoxide (BS087-100 ml, Biosharp, China) was applied to the lower left part of the back of the mice. For the GSH function experimental group, dorsal shaved mice were intraperitoneally injected with 100 mg/kg (body weight) of reduced GSH (G8180, Solebo, China) and 450 mg/kg (body weight) of BSO (HY-106376, MCE, China), while the control group was treated by phosphate-buffered saline (PBS) (T8166, Taojiao, China) or dimethyl sulfoxide (BS087-100 ml, Biosharp, China). In addition, 50 μl of 60 μM XN (T3342, Taojiao, China), 10 mM 3-Methyladenine (HY-19312, MCE, China), and PBS (BL601A, Biosharp, China) were injected into the right dorsal side of C57 mice after plucking at 8 weeks of age [21]. For the shaved group, the drug was administered every other day for a total of 5 times, followed by observation of pigmentation on the backs of the mice. When gray coloration was observed on the backs of the mice, the skin of the backs of the mice was taken and embedded or preserved at −80°C for qRT-PCR or western blot. For the plucked group, the drug was administered every day, and the skin of the backs of the mice was taken and embedded or preserved at −80°C on the third day and the sixth day after the plucking of the hairs for qRT-PCR or western blot. The small-molecule inhibitors and activators are included in Table S1.

### BrdU administration

To label transiently multiplying cells with BrdU, mice were intraperitoneally injected with 50 mg/kg (body weight) of BrdU (Beyotime, Sigma). Samples were collected at 4 h of intraperitoneal injection, and transiently multiplying cell expression was observed using a fluorescent primary antibody to BrdU (MAB3222, Chemicon, USA). The green fluorescence of BrdU was observed under the excitation light of 488 nm to costain with nuclei and the number of BrdU^+^ cells was counted.

### Real-time quantitative reverse transcription PCR

The skin tissues from the back of mice were removed and stored at −80°C. Then it was ground in liquid nitrogen, 1 ml of TRIzol (NR0002, Regen, China) was added to the ground sample for lysis, chloroform was added to dissociate the nucleoprotein complex completely, the upper aqueous layer was transferred to isopropanol after centrifugation, and the upper aqueous layer was added to 70% ethanol after centrifugation and washed twice. The lid was opened to volatilize off the excess ethanol, which is then added to diethyl pyrocarbonate water (NR0001, Regen, China) to be stored in −80°C. Next, the extracted RNA was reverse transcribed into cDNA using a reverse transcription kit (RT-01023, FOREG ENE, China). qRT-PCR was carried out on a Hongshi SLAN-96P instrument after adding primers and TB Green Premix Ex Taq (2X) (RR420L, Takara, Japan) to the 10-fold diluted cDNA. All reactions were repeated for 3 wells and averaged. The prime sequences in PCR are included in Table S2.

### H&E staining

Mouse dorsal skin tissues were cut and soaked in 4% paraformaldehyde, placed at 4°C for 48 h, dehydrated and embedded in paraffin, and sectioned to make tissue samples. The samples were stained with H&E for 1.5 min at room temperature and differentiation solution for 3 min, then immersed in tap water for 15 min, dehydrated with xylene, and fixed with neutral resin. Finally, histopathologic examination was performed with a phase contrast microscope (Mito, China) and photographed with NScope 2.0.

### Immunofluorescence staining

Mouse dorsal skin tissues were clipped, immersed in 4% paraformaldehyde, and placed at 4°C for 48 h. The samples were dehydrated and embedded in paraffin and sliced into sections of 10 μm in thickness. Sections were baked in an oven at 65°C for 20 min, followed by immersion in xylene for dewaxing, hydration using gradient ethanol, and antigen repair with citric acid (#C805019, Macklin, China) and sodium citrate solution (#S818273, Mackling, China). The antigen-repaired sections were then closed using 2% bovine serum albumin solution (#A8020, Solarbio, China) in an oven at 37°C for 1 h. Primary antibodies were then added and incubated at 4°C overnight. The samples were rewarmed at room temperature for 2 h. The primary antibody was washed off. The slides were incubated with fluorescently labeled secondary antibodies (Alexa Fluor 488-conjugated goat anti-mice immunoglobulin G, Beyotime, China or Alexa Fluor 488-conjugated goat anti-mice immunoglobulin G, Beyotime, China) for 2 h at 37°C and 4′,6-diamidino-2-phenylindole (DAPI) (#c-1002, Beijing, China) for half an hour at room temperature, and finally, the slides were sealed with antifluorescent quencher and nail polish. Images were taken under a laser confocal microscope (Lecia, Germany) in the Oncology Laboratory of the Affiliated Cancer Hospital of Chongqing University. The relative fluorescence intensity of the image was first divided into 8-bit black-and-white images of red, green, and blue channels using ImageJ software. Then, the fluorescence intensity of each channel was calculated separately. Finally, the ratio of the fluorescence intensity of the target protein to the fluorescence intensity of the nucleus was calculated to obtain the relative fluorescence intensity. The antibodies are included in Table S3.

### Transmission electron microscopy

The back skin of mice was soaked in 4% glutaraldehyde at a temperature of 4°C and fixed overnight. The back skin was rinsed 3 times in sodium bicarbonate buffer. Then, the back skin was rinsed 3 times with distilled water and expose it to 1% uranyl acetate water at room temperature for 15 min. The back skin was rinsed twice in distilled water. Dehydration treatment was performed on the dorsal skin using a gradient ethanol solution. The embedding agent penetrates the embedded tissue. The dorsal skin tissue was polymerized at 36 to 60°C, ultrathin sections were made, cells from hair follicles were selected, and they were collected on a copper mesh. The grid was poststained in toluidine blue. Slices were observed in a Philips CM-10 transmission electron microscope (FEI Italia, 20122 Milan, Italy), and micrographs were recorded on Kodak 4489 film.

### Reduced GSH content assay

We used the Reduced GSH Content Assay Kit (#BC1170, Solarbio, China) to detect the differences in GSH content in different treated samples. The skin tissues from the back of mice were removed and weighed, according to 0.1 g of tissue, 1 ml of reagent I in liquid nitrogen and grind. The ground sample was placed in a centrifuge, 8,000g, 4°C centrifugation for 10 min, and then the supernatant was taken and put at 4°C. The enzyme counter was preheated for more than 30 min, and the wavelength was adjusted to 412 nm. Standard solution (10 mg/ml) was aspirated and diluted to 300, 200, 100, 50, and 25 μg/ml with distilled water, and the assay tubes, the standard tubes, and the blank tubes were set up following the procedure of the manual, respectively. Then, the standard curve was plotted according to the absorbance of the standard tube. According to the mass of the sample, the GSH content of the sample was calculated by substituting the formula.

### Mitochondrial probe staining

We used a mitochondrial red fluorescent probe (C1049B-50 μg, Biosharp, China) on hair follicles to label biologically active mitochondria in the hair follicle. Firstly, after collecting the dorsal skin of the mice, the samples were embedded with cryosection embedding agent (#SAKURA-4583, Biosharp, China) at −80°C, and then the samples were sliced with a cryoslicer into 10-μm-thick slices for probe staining. One microliter of 200 μM Mito-Tracker Red CMXRos storage solution was added to 1 ml of e.g. Hank’s balanced salt solution containing calcium and magnesium ions, and mixed well to make 200 nM Mito-Tracker Red CMXRos working solution. The frozen sections were washed with PBS to wash off the embedding agent, and an appropriate amount of 200 nM Mito Tracker Red CMXRos Working Solution was added to the section samples and incubated at 37°C for 30 min. After removing the Mito-Tracker Red CMXRos working solution, DAPI was added and incubated at room temperature for 30 min; then, the slides were sealed with an antifluorescence quencher. Finally, the samples were examined for the intensity of the red fluorescence in the wavelength range of 579 to 599 nm under a confocal microscope.

### Western blot

Mouse skin was ground into powder under liquid nitrogen, and an appropriate amount of protein lysis solution radioimmunoprecipitation assay and an equal proportion of protease inhibitor PMSF were added. The working solution in the BCA protein concentration determination kit (P0012, Beyotime, China) was used to measure the absorbance of the sample at 562 wavelengths, and the sample concentration was calculated based on the standard curve. Next, the required sample size for each sample was calculated based on the sample concentration. After adding the sample, electrophoresis was carried out at 60 V for 30 min, then at 120 V. Electrophoresis was stopped when bromophenol blue reaches the bottom of the gel. Then, at low temperature, the protein was transferred onto the polyvinylidene difluoride membrane under a constant current of 200 mA for 2 h and was sealed in 10% skim milk powder for 1 h. After washing the milk powder on the membrane with TBST, the polyvinylidene difluoride membrane was cut according to the size of the marker and the target protein. The cut membrane was put into an antibody box, and the primary antibody was added. The membrane was incubated overnight in a refrigerator at 4°C. The primary antibody liquid on the TBST membrane was washed and incubated in the secondary antibody for 2 h. Finally, observations were made and recorded on the ECL luminescent instrument.

### Skin organoid culture

The method for primary newborn mice cell culture can be referred to our previous publication [21]. Cells were extracted from the dorsal skin of newborn mice within 24 h after birth, and the dermis and epidermis were separated by floating the dorsal skin in 0.25% trypsin (#150057, Gibco, USA) solution at 4°C overnight. Epidermal cells were obtained by shearing, filtration, and centrifugation. Dermal cells were digested in 0.35% collagenase I (#LS004197, Worthington, USA) for 20 min and then obtained by filtration and centrifugation. Isolated epidermal and dermal cells were mixed in a 1:9 ratio and dropped into the upper chamber of transwell cultures, and the lower chamber was filled with 700 μl of Dulbecco’s modified eagle medium/F12 (#MT100 13CV, Corning, USA) medium (#1099-141C, Gibco, USA) containing 10% fetal bovine serum. Cells were cultured in a 5% CO_2_ incubator at 37°C, and the medium was changed every day.

### Transplantation

Nude mice were covered with betaine solution under anesthesia. The skin on the back and both sides of the back was cut (1-cm diameter). Cell cultures were transplanted onto the backs of nude mice. Two weeks after transplantation, the bandages were removed, and regenerating hair follicles were photographed and counted.

### RNA interference

We transfected shRNA into extracted epithethial cells using Lipofectamine 3000 (#L3000008, Thermo Fisher, USA) according to the manufacturer’s instruction. Briefly, Lipofectamine 3000 reagent was diluted in 125 μl of Opti-MEM medium (#31985062, Thermo Fisher, USA). P3000 (10 μl) and shRNA (4 μg) were separately diluted in Opti-MEM medium and incubated at room temperature for 15 min. The DNA and P3000 mixtures were then combined and added dropwise to the epidermal cells for a 6-h incubation. After 48 h, qRT-PCR was used to evaluate the KD efficiency of each shRNA sequence. The shRNAs with the best KD efficiency were used in subsequent experiments either by transfection or packaged into adenoviruses for in vivo delivery. To deliver shRNA via adenoviral vectors, 1 × 10^8^ PFU of adenoviral particles (Ad-shBhlhe40 or Ad-shSox9) in 40 μl were injected subcutaneously into the exposed dorsal skin 2 d before, on the day of, and 2 d after hair plucking. Samples were collected and analyzed on PPD3 and PPD6. The shRNA sequences are listed in Table S4.

### Bulk RNA-seq analysis

RNA-seq data for hair follicles of telogen and early anagen (GSE86955) were downloaded from the Gene Expression Omnibus database. For clustering analysis, differentially expressed genes among hair follicles of telogen and early anagen were determined by limma. False discovery rate < 0.05 and log2 fold change > 1 were used as a threshold to determine significant differences in gene expression. Differential expression gene enrichment and functional annotation analysis were performed by the David database.

### ScRNA-seq analysis

The skin of mice at 3 and 4 weeks were collected for scRNA-seq. We used Seurat v.4.2.2 to perform QC, normalization, feature selection, linear and nonlinear dimensional reduction, cell clustering, finding cluster biomarkers, and assigning cell type identity to clusters. Clusters “Bu”, “HG”, and “DP” were divided by specified markers: CD34, Krt5, Ccnd1, Lhx2, Enpp2, and Wif1 (Fig. 1D). We selected the “HG” clusters for further analysis. KEGG enrichment analysis (Fig. 5D) was performed by https://www.bioinformatics.com.cn, an online platform for data analysis and visualization. ScRNA-seq data of the hair follicles for 18 months was obtained from GSE227784.

### Statistical analyses

Data were collected from 3 independent experiments and expressed as mean ± standard deviation (SD). All significance comparisons, and *t* tests were performed using Origin 8.0 software. *P* values were considered significant at 0.05, 0.01, and 0.001 levels.

## Data Availability

Bulk RNA-seq data of hair follicles at telogen on postnatal day 47 and early anagen on postnatal days 29 to 31 were acquired from GSE67404. ScRNA-seq data of the hair follicles of 5 and 9 weeks were obtained from GSE129218. Bulk RNA-seq data for skin of young mice (2 months old) and aged mice (18 months old) were obtained from GSE185087. ScRNA-seq data of the 18-month-old hair follicles were obtained from GSE227784.
