# Supplementary material for: Mitophagy Promotes Hair Regeneration by Activating Glutathione Metabolism
Source: Research (Wash D C). 2024 Aug 1;7:0433. doi: 10.34133/research.0433 (PMC11292124; doi:10.34133/research.0433)
Supplement: Supplementary 1 — Figs. S1 to S7 Tables S1 to S4 [file research.0433.f1.pdf]

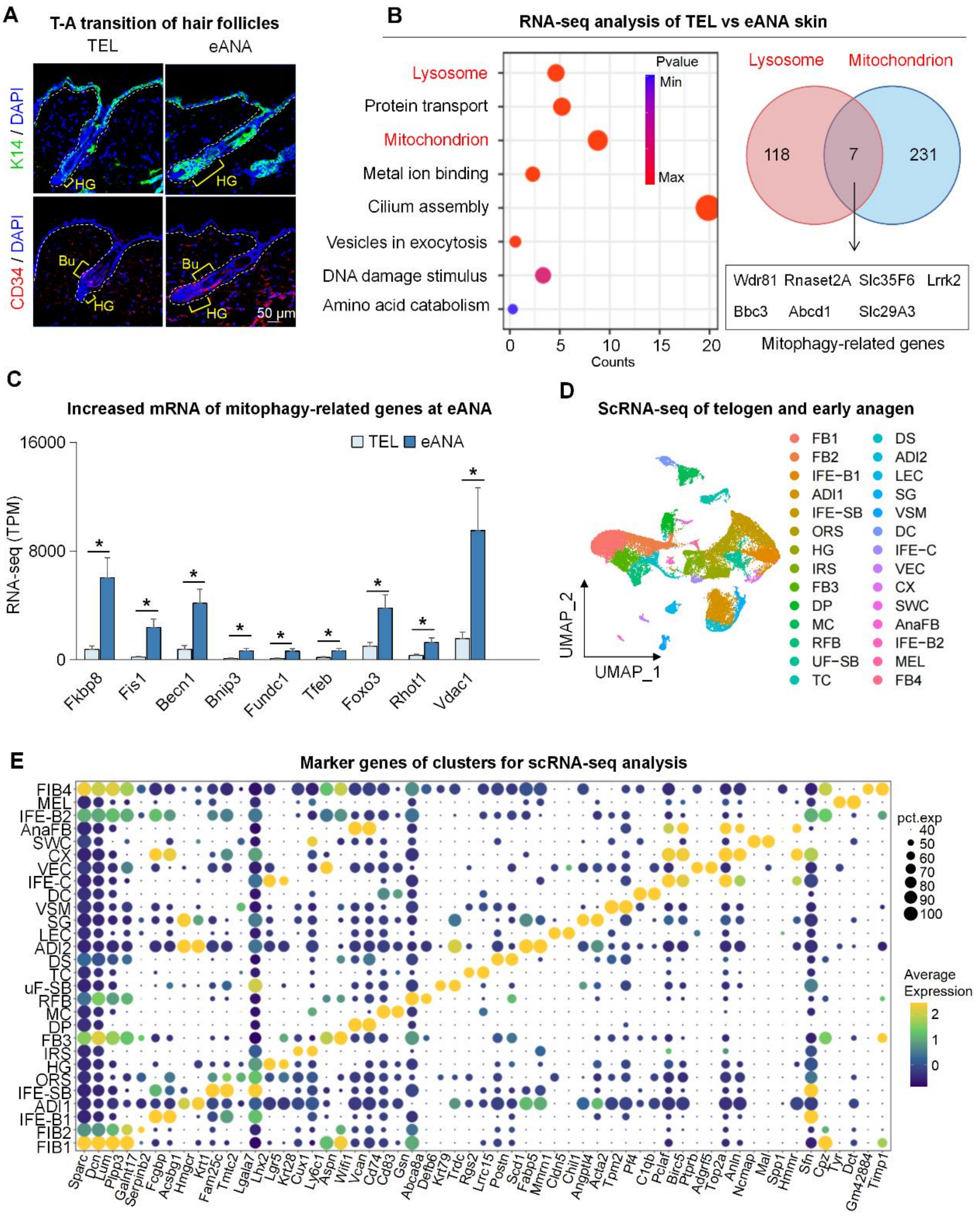

Fig. S1. Increased mitophagy at early anagen

A. K14 and Cd34 immunostaining of hair follicles show the transition from telogen to early anagen. Scale bars, 50 μm.

B. RNA-seq compares gene expression between telogen and early HFSCs. KEGG analysis shows the lysosome and mitochondrion signaling pathway enriched in differentially expressed genes (DEGs) of telogen and early HFSCs (left); Venn diagram shows genes co expressed by lysosome and mitochondrion pathways (right).

C. RNA-seq compares the gene expression of mitophagy pathways in HFSCs

between telogen and early anagen. N=3, *p<0.05.
UMAP plots of 24 clusters by unbiased clustering.

D. Dotplot of marker genes of 24 clusters.

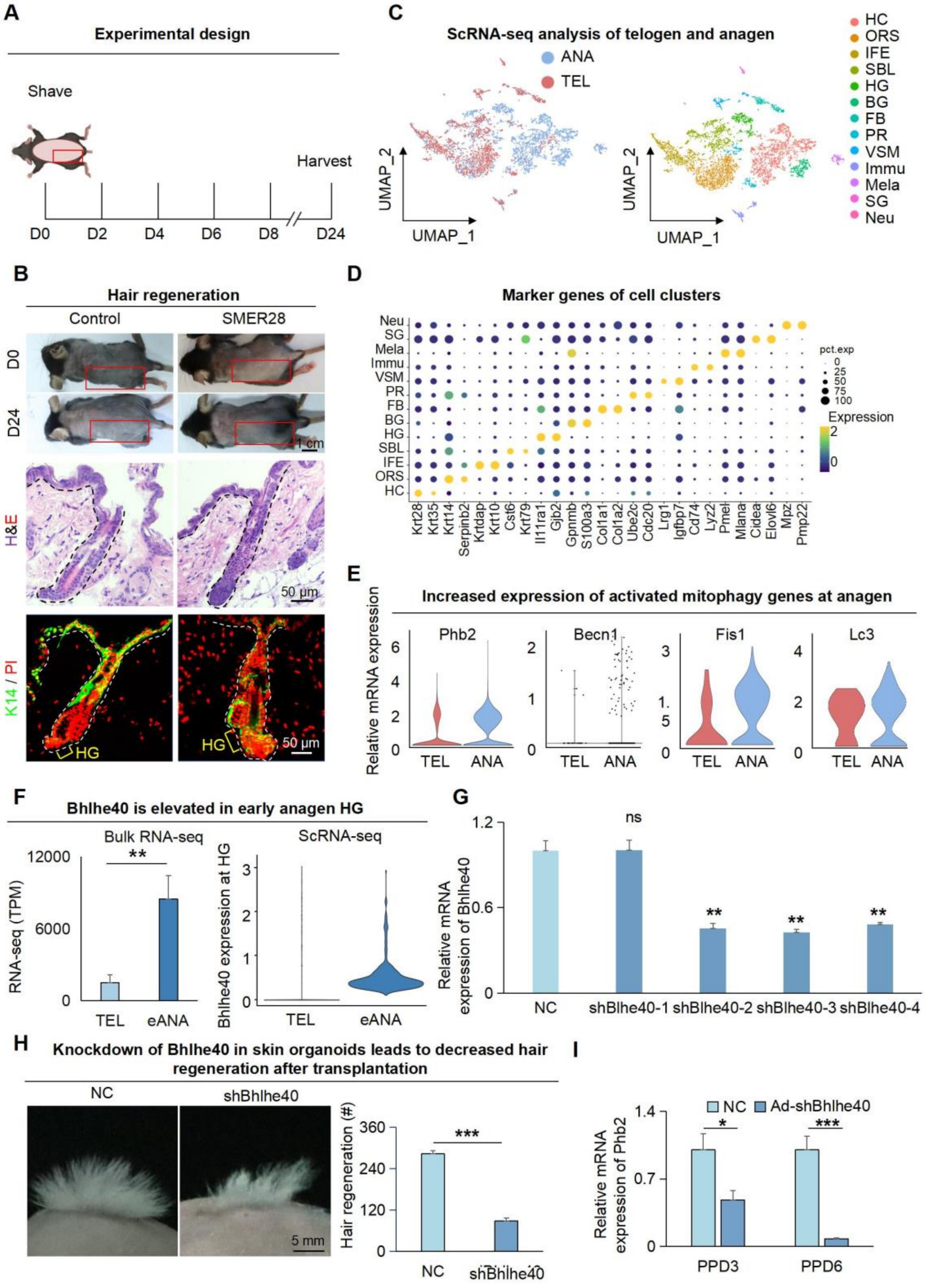

Fig. S2. Increased expression of Phb2 at the hair germ during anagen

A. Schematic of experimental design.

B. Photographs, HE staining and K14 immunostaining of hair follicles show that SMER28 induce hair regeneration. Scale bars, 50 μm.

C. Cell clustering of hair follicles of combined 5 weeks (ANA) & 9 weeks (TEL) cultures.

D. Dotplot of marker genes of 13 clusters.

E. VlnpIot shows the gene expression of mitophagy pathways between telogen and anagen.
VlnPlot shows the gene expression of mitophagy pathways between telogen and anagen.

F. Bulk RNA-seq and VlnPlot shows the expression of Bhlhe40 at early anagen HG.

G. Quantitative RT-PCR shows the knockdown of Bhlhe40 after treatment with various shRNA sequences. (NC represents non-targeting control; shBhlhe40-1, shBhlhe40-2, shBhlhe40-3, and shBhlhe40-4 target distinct RNA sequences to knockdown of Bhlhe40). N=3, ns $p>0.05$, **$p<0.01$.

H. Left: Representative images illustrating hair regeneration after skin organoid transplantation from the NC and shBhlhe40 groups. Right: Quantitative analysis of the number of hair follicles regenerated. n=3, ***$p<0.001$.

I. Quantitative RT-PCR shows the expression of Phb2 after shRNA knockdown of Bhlhe40 in vivo. N=3, *$p<0.05$, ***$p<0.001$.

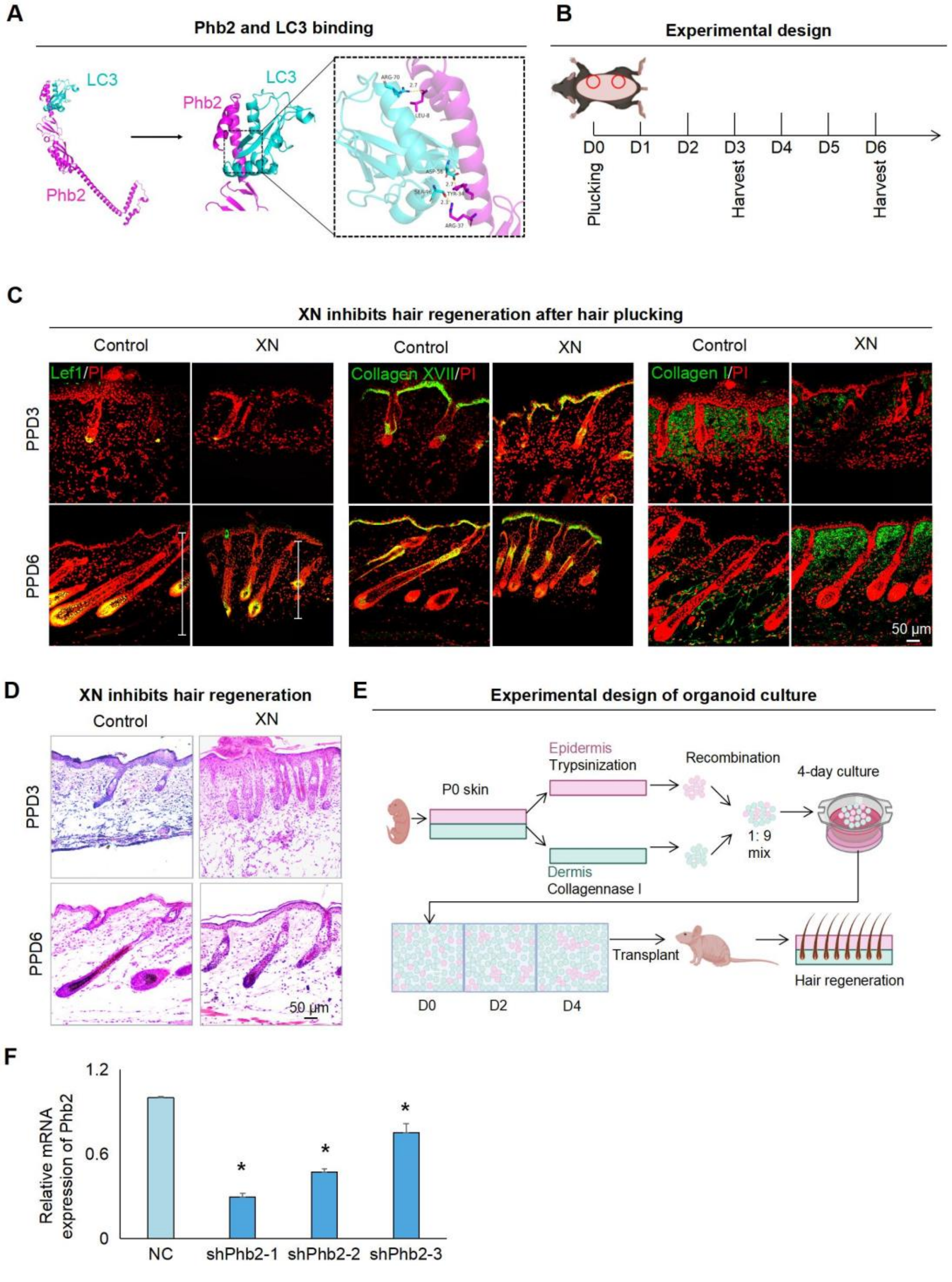

Fig. S3. Inhibition of Phb2 and Lc3 binding by Xanthohumol inhibits hair follicle regeneration

A. PyMOL displays the molecular docking results of Lc3 and Phb2

B. Schematic of experimental design.

C. Collagen I, Collagen XVII and Lef1 immunostaining of hair follicles show that XN inhibits hair regeneration at PPD3 and PPD6. Scale bars, 50 μm.

D. HE staining shows that XN inhibits proliferation of hair germ cells at PPD3 and PPD6. Scale bars, 50 μm.

E. Schematic of experimental design of organoid culture.

F. Quantitative RT-PCR analysis of Phb2 expression upon knockdown in epidermal cells (NC represents non-targeting control; shPhb2-1, shPhb2-2, and shPhb2-3 target distinct DNA sequences to knockdown of Phb2). N=3, *$p<0.05$ compared to NC.

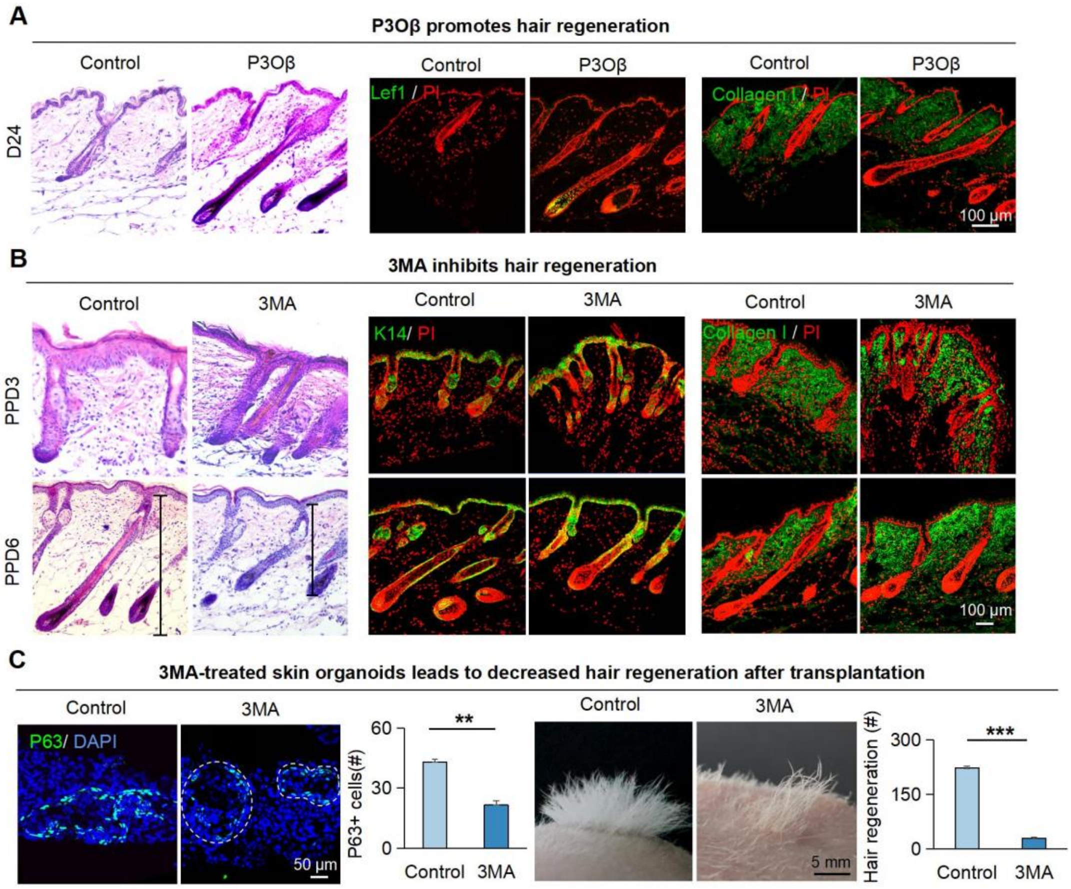

Fig. S4. Lc3 mediated mitophagy to promote hair follicle regeneration

A. HE staining, Collagen I and Lef1 immunostaining of hair follicles show that P3Oβ induce hair regeneration. Scale bars, 50 μm.

B. HE staining, Collagen I and K14 immunostaining of hair follicles show that 3MA Inhibits hair regeneration at PPD3 and PPD6. Scale bars, 50 μm.

C. 3MA in skin organoids inhibits hair regeneration after transplantation. P63 immunostaining of skin organoid cultures of newborn mice cells shows the epidermal stem cells; Photographs and statistical analysis show hair regeneration after grafting. Scale bars, 50 μm and 5 mm. N=3, ***p<0.001, **p<0.01.

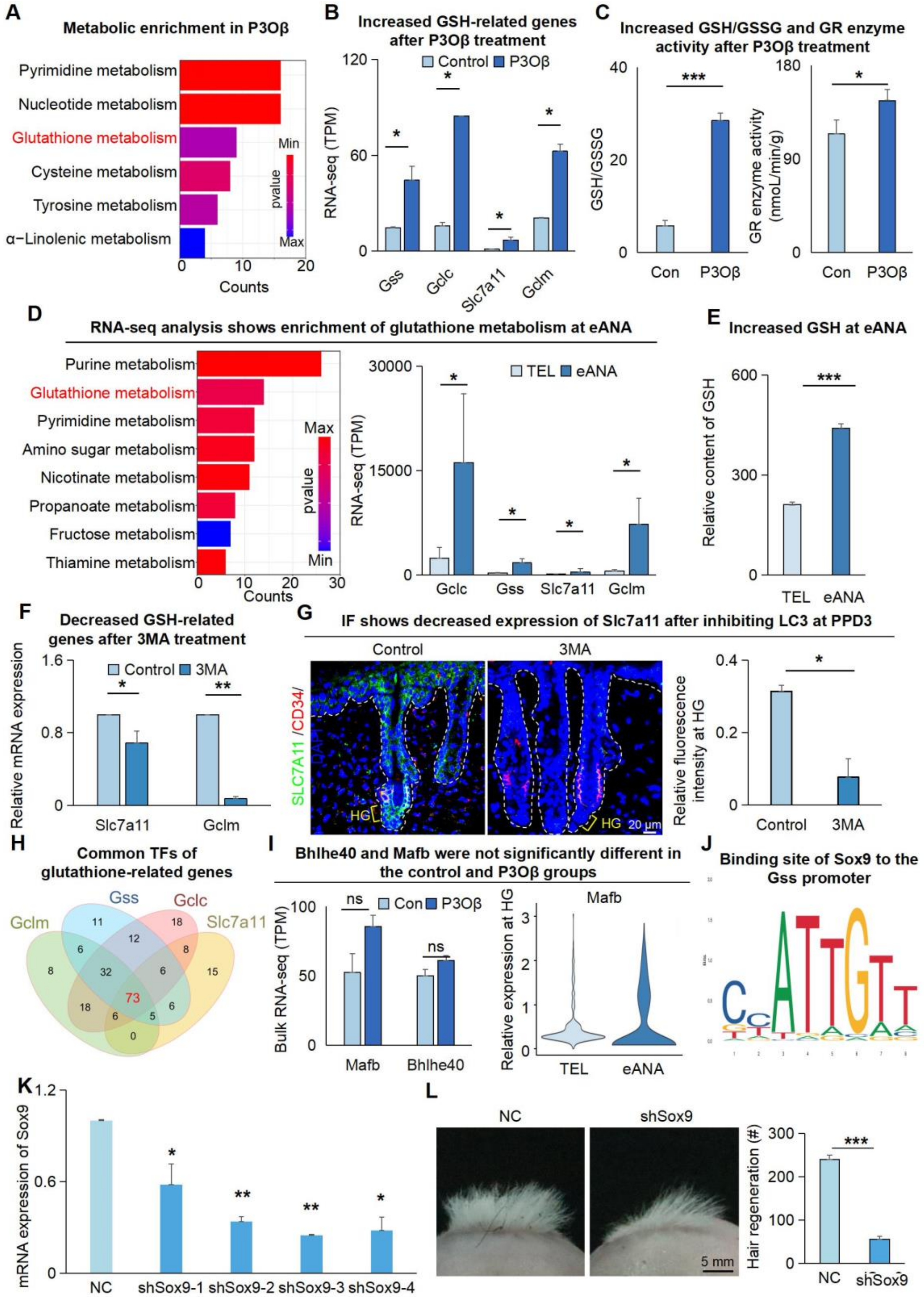

Fig. S5. Lc3 mediated mitophagy regulates glutathione metabolism pathway

A. RNA-seq compares gene expression in hair follicles between control and P3Oβ-treated groups. KEGG analysis shows the glutathione metabolism enriched in differentially expressed genes (DEGs) of control and P3Oβ-treated groups.

B. RNA-seq shows gene expression of glutathione metabolism pathway in hair

follicles between control and P3Oβ-treated groups. N=3, *p<0.05.

C. GSH and GSSG assay kits showed that the GSH/GSSG content in P3Oβ was significantly increased (left); GR assay kits showed that the GR enzyme activity in P3Oβ was significantly increased. N=4, ***p<0.001, *p<0.05.

D. RNA-seq shows gene expression of glutathione metabolism pathway in hair follicles between telogen and early anagen. KEGG analysis shows the glutathione metabolism enriched at early anagen (left). RNA-seq compares the gene expression of glutathione metabolism pathway in HFSCs between telogen and early anagen. N=3, *p<0.05.

E. The glutathione assay kit determined the glutathione content of telogen and early anagen. N=3, ***p<0.001.

F. Quantitative RT-PCR shows the mRNA expression of glutathione metabolism pathway that are differentially expressed at the control and 3MA-treated groups. N=3, **p<0.01, *p<0.05.

G. Immunofluorescence of Slc7a11 shows the expression in the control group and 3MA-treated group. Scale bars, 50 μm. N=3, *p<0.05.

H. Venn diagram shows the number of TFs predicted by four genes related to glutathione synthesis.

I. Bulk RNA-seq displays the expression of Mafb and Bhlhe40 in the control group and P3Oβ group (left). VlnPlot shows that the mRNA expression of Mafb is differentially expressed between telogen and early anagen HG (right). N=3, ns, p>0.05.

J. The binding site of Sox9 to the Gss promoter sequence.

K. Quantitative RT-PCR shows the expression of Sox9 when knockdown of Sox9. (NC represents negative control; shSox9-1, shSox9-2, shSox9-3, and shSox9-4 target distinct RNA sequences to knockdown of Sox9). N=3, *p<0.05, **p<0.01.

L. Left: Representative images illustrating hair regeneration after skin organoid transplantation from the NC and shSox9 groups. Right: Quantitative analysis of the number of hair follicles regenerated. n=3, ***p<0.001.

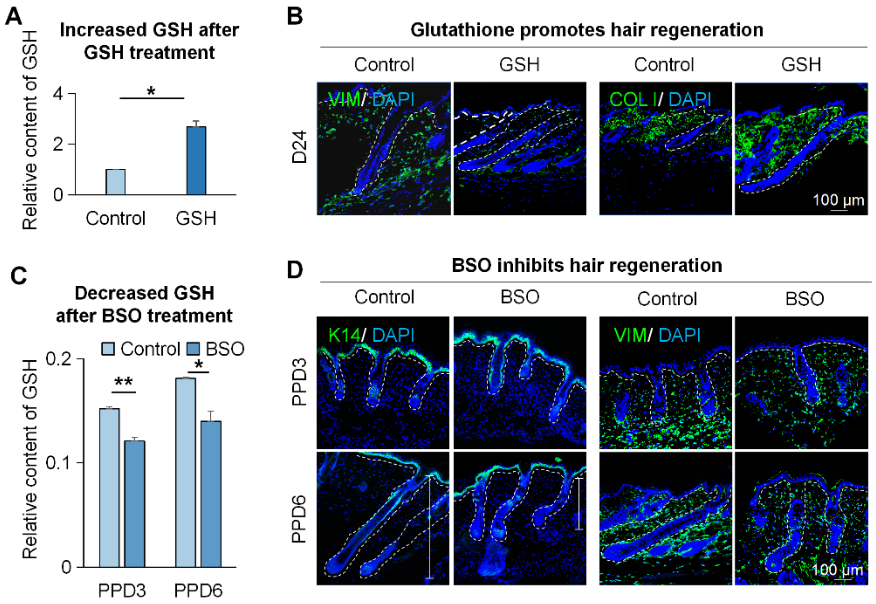

Fig. S6. Glutathione promotes hair follicle regeneration

A. The glutathione assay kit determined the glutathione content of control and GSH group. N=3, *$p<0.05$.

B. Vimentin and Collagen I immunostaining of hair follicles show that GSH induce hair regeneration. Scale bars, 100 μm.

C. The glutathione assay kit determined the glutathione content of control and BSO group at PPD3 and PPD6. N=3, **$p<0.01$, *$p<0.05$.

D. K14 and Vimentin immunostaining of hair follicles show that BSO Inhibits hair regeneration at PPD3 and PPD6. Scale bars, 100 μm.

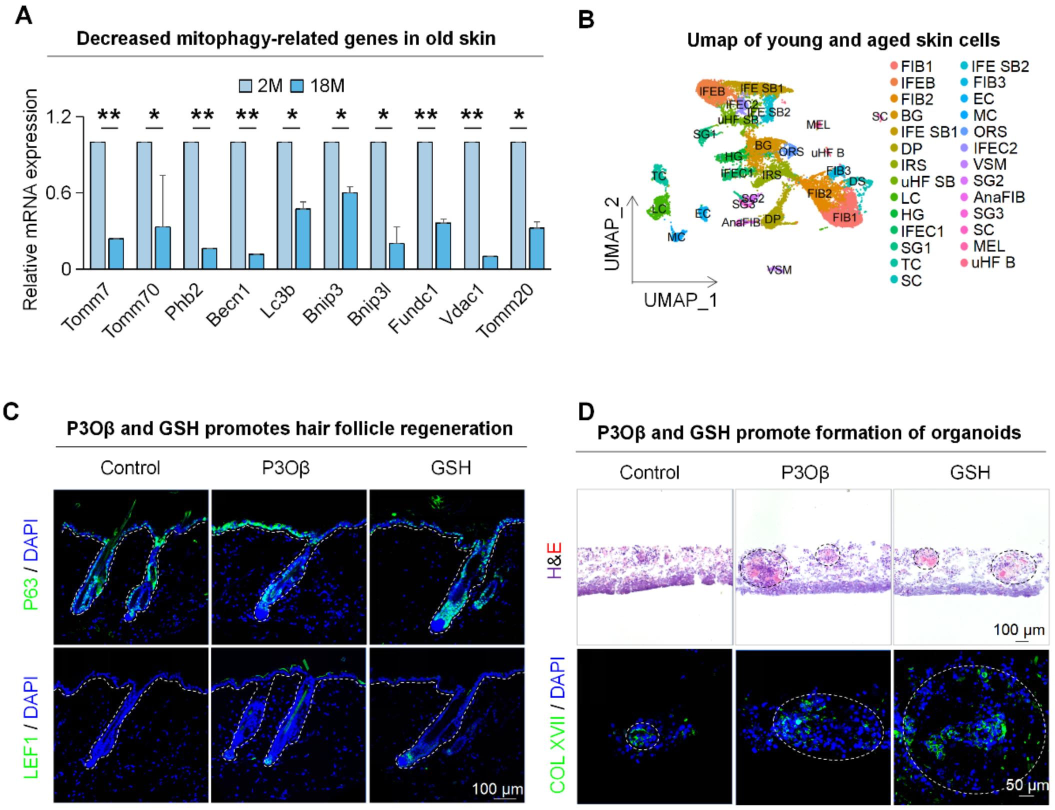

Fig. S7. P3Oβ and glutathione promote hair follicle regeneration in aged mouse

A. Quantitative RT-PCR shows the mRNA expression of mitophagy pathway that are differentially expressed in young and old mice. N=3, $^{**}p<0.01$, $^{*}p<0.05$.

B. Cell clustering of hair follicles of combined 2 months (young) & 18 months (old) cultures.

C. P63 and Lef1 immunostaining of hair follicles show that P3Oβ and GSH induce hair regeneration in old. Scale bars, 100 μm.

D. HE staining and Collage XVII shows the formation of aggregates after treatment of P3Oβ and GSH. Scale bars, 100 μm.

Table S1. Small molecule inhibitors and activators in this study.

Product Description	Company	Cat#
SMER28 (autophagy activator)	Topscience	T3155
Pennogenin 3-O-beta-chacotrioside (LC3 activator)	Topscience	T8657
KYP-2047 (BECN1 activator)	Topscience	T8166
GSH	MCE	HY-D0187
BSO (GSH inhibitor)	MCE	HY-106376
3MA (LC3 inhibitor)	MCE	HY-19312
Xanthohumol (Phb2 ligand)	MCE	HY-N1067
BrdU	Beyotime	ST1056

Table S2. Sequences of the Real-time qPCR.

Gene name	Forward primer (5'-3')	Reverse primer (5'-3')
Becn1	ATGGAGGGGTCTAAGGCGTC	TCCTCTCCTGAGTTAGCCTCT
Prkn	TCTTCCAGTGTAACCACCGTC	GGCAGGGAGTAGCCAAGTT
Bnip3	TCCTGGGTAGAACTGCACTTC	GCTGGGCATCCAACAGTATTT
Fundc1	TGTGATATCCAGCGGCTTCG	GCCGGCTGTTCCTTACTTTG
Vdac1	GTGCTCTGGTGCTGGGTTAT	CTCCGTTCCGTCGTTCACAT
Bnip3l	TTGGATGCACAACATGAATCAGG	TCTTCTGACTGAGAGCTATGGTC
Phb2	ATCCGTGTTCACCGTGGAAG	CCCGAATGTCATAGATGATGGG
Lc3b	TTATAGAGCGATACAAGGGGGAG	CGCCGTCTGATTATCTTGATGAG
Pink1	GGACTCAGATGGCTGTCCCT	ATTGCCACCACGCTCTACAC
Tomm20	AATGCCATTGCTGTGTGTGG	GGTCGGAAGCTTGGTCAGAA
Tomm7	ATCCGCTGGGGCTTTATTCC	CGACGGTTCAGGCATTCCA
Tomm70a	TGGGGCTATGTACCTGTGGAG	GCTTGCTCGTATTTTCCTGCTT
Gclc	GGGGTGACGAGGTGGAGTA	GTTGGGGTTTGTCCTCTCCC
Gss	CAAAGCAGGCCATAGACAGGG	AAAAGCGTGAATGGGGCATAC
Slc7a11	GGTCCATTACCAGCTTTTGTACG	AATGTAGCGTCCAAATGCCAG
Gclm	CATTTACAGCCTTACTGGGAGG	ATGCAGTCAAATCTGGTGGCA
Gapdh	TGGCCTTCCGTGTTCCTAC	GAGTTGCTGTTGAAGTCGCA

Table S3. Antibodies used in this study.

Antibody	Isotype	Company	Cat#
Cd34	Rat	Invitrogen	14-0341-82
Fundc1	Rabbit	bioss	bs-13227R
PCNA	Mouse	Elabscience	E-AB-22001
Vimentin	Mouse	Beyotime	AF0318
P63	Rabbit	GeneTex	GTX102425
Lef1	Rabbit	zenbio	380956
LC3	Rabbit	Proteintech	14600-1-AP
Laminin	Rabbit	Abcam	ab11575
K14	Rabbit	Boster	A01432
Anti-BrdU	Mouse	Chemicon	MAB3222
Beclin1	Rabbit	zenbio	381896
Collagen I	Goat	Arigobio	ARG21965
Collagen XVII	Rabbit	Beyotime	AF1078
GCLC	Rabbit	Beyotime	AF6969
GSS	Rabbit	Beyotime	AF7037
PHB2	Rabbit	BOSTER	A03315-3
SLC7A11	Rabbit	Beyotime	AF7992
Sox9	Mouse	Proteintech	67439

Table S4. The target sequence of shRNA.

Gene	Target sequence
Bhlhe40	AATTGCAGTGGTTCTGGAGCTTACGCTCGAGCGTAAGCTCCAGAACCACTGCTTTTTT
Phb2	AATTGAAGGAGAGCGAGGAAGATAACTCGAGTTATCTTCCTCGCTCTCCTTCTTTTTT
Lc3	AATTGCTCAATGCTAACCAAGCCTTCTCGAGAAGGCTTGGTTAGCATTGAGCTTTTTT
Sox9	AATTGAAGGAGAGCGAGGAAGATAACTCGAGTTATCTTCCTCGCTCTCCTTCTTTTTT
